# Multi-population genomic analysis of malaria parasites indicates local selection and differentiation at the *gdv1* locus regulating sexual development

**DOI:** 10.1038/s41598-018-34078-3

**Published:** 2018-10-25

**Authors:** Craig W. Duffy, Alfred Amambua-Ngwa, Ambroise D. Ahouidi, Mahamadou Diakite, Gordon A. Awandare, Hampate Ba, Sarah J. Tarr, Lee Murray, Lindsay B. Stewart, Umberto D’Alessandro, Thomas D. Otto, Dominic P. Kwiatkowski, David J. Conway

**Affiliations:** 1 Pathogen Molecular Biology Department, London School of Hygiene and Tropical Medicine, Keppel St, London, UK; 2 MRC Gambia Unit, Fajara, The Gambia; 3 Le Dantec Hospital, University Cheikh Anta Diop, Dakar, Senegal; 4 Malaria Research and Training Center, University of Bamako, Bamako, Mali; 5 West African Centre for Cell Biology of Infectious Pathogens (WACCBIP) and Department of Biochemistry, Cell and Molecular Biology, University of Ghana, Legon, Ghana; 6 Institut National de Recherches en Santé Publique (INRSP), Nouakchott, Mauritania; 7 Malaria Programme, Wellcome Trust Sanger Institute, Cambridge, UK; 8 Disease Control Department, London School of Hygiene and Tropical Medicine, Keppel St, London, UK

## Abstract

Parasites infect hosts in widely varying environments, encountering diverse challenges for adaptation. To identify malaria parasite genes under locally divergent selection across a large endemic region with a wide spectrum of transmission intensity, genome sequences were obtained from 284 clinical *Plasmodium falciparum* infections from four newly sampled locations in Senegal, The Gambia, Mali and Guinea. Combining these with previous data from seven other sites in West Africa enabled a multi-population analysis to identify discrete loci under varying local selection. A genome-wide scan showed the most exceptional geographical divergence to be at the early gametocyte gene locus *gdv1* which is essential for parasite sexual development and transmission. We identified a major structural dimorphism with alternative 1.5 kb and 1.0 kb sequence deletions at different positions of the 3′-intergenic region, in tight linkage disequilibrium with the most highly differentiated single nucleotide polymorphism, one of the alleles being very frequent in Senegal and The Gambia but rare in the other locations. Long non-coding RNA transcripts were previously shown to include the entire antisense of the *gdv1* coding sequence and the portion of the intergenic region with allelic deletions, suggesting adaptive regulation of parasite sexual development and transmission in response to local conditions.

## Introduction

The malaria parasite *Plasmodium falciparum* has evolved in response to diverse environments, including different human and mosquito host populations throughout most of the world^1,2^, and changes caused by drug treatment and other malaria control efforts^3^. It is vital to understand this species as it is responsible for approximately half a million human deaths every year, mostly in Africa^4^. The parasite exhibits significant population genetic substructure within Asia and South America, due to geographical isolation of some endemic areas, and emergence of local lineages carrying different drug resistance alleles under recent selection^5,6^. In contrast, there is high local diversity and minimal divergence among populations in Africa^7^, particularly within West Africa^6,8–10^ where malaria is endemic in all areas south of the Sahara^11^.

Genome-wide analyses have identified parasite loci under differential local selection pressures in Southeast Asia, where resistance has emerged to many antimalarial drugs, most recently to artemisinin derivatives and piperaquine^12^. Within Africa, frequencies of genotypes conferring resistance to previously used antimalarial drugs has been determined by local history of drug selection^13^ and regional dissemination of alleles^14^. While the role of drug selection is well recognised, the importance of other processes involved in local adaptation remains relatively unknown.

A few genome-wide comparisons have been performed on pairs of local populations from different African countries^9,10^, or within a single country^8^. In the first study, parasites in an area of The Gambia with a moderate level of seasonal transmission were compared with those in an area of more continuous high level transmission in Guinea, indicating higher divergence in SNPs in or around the *gdv1* gene on parasite chromosome 9 than at any other part of the genome^10^. This observation was noted to be of interest as the *gdv1* gene is the earliest marker of parasite differentiation from asexual replicating blood stages into sexual stage gametocytes which are transmitted to mosquitoes^15,16^. In areas with limited seasonal transmission, parasites might be able to detect when conditions become favourable in order to increase gametocyte production^17^, and this could potentially involve regulation of *gdv1* expression. However, population genomic data from Mauritania where transmission is limited to a short season in comparison with the highly endemic population in Guinea did not show differentiation at the locus^9^.

Comparison of these initial studies indicates that broad systematic comparisons of multiple populations are required to provide an unbiased scan for loci under locally varying selection. To enable a large multi-population analysis here, new population samples of *P. falciparum* infections from four different areas in West Africa were sequenced, and results analysed together with data from seven other areas in this diverse endemic region. Signatures of locally varying selection are clearly seen at multiple loci, and the most geographically divergent genomic locus is the *gdv1* gene and the large 3′-adjacent intergenic sequence. Focusing on this locus, we identify a major dimorphism comprising large mutually exclusive deletions in the intergenic sequence, one of the alleles being at high frequency in The Gambia and Senegal but uncommon elsewhere in West Africa. Transcription of the *gdv1* locus in a cultured laboratory parasite line was previously shown to be dominated by long non-coding RNA, with spliced transcripts of parts of the intergenic sequence and the entire antisense of the gene^18,19^. Identification of an important locus for parasite sexual stage development as being under local selection makes it a priority to uncover mechanisms of gene regulation and explore its potential as a target for blocking transmission of infection in different endemic environments.

## Results

### Genome-wide single nucleotide polymorphism data from genome sequences

**Figure 1:**
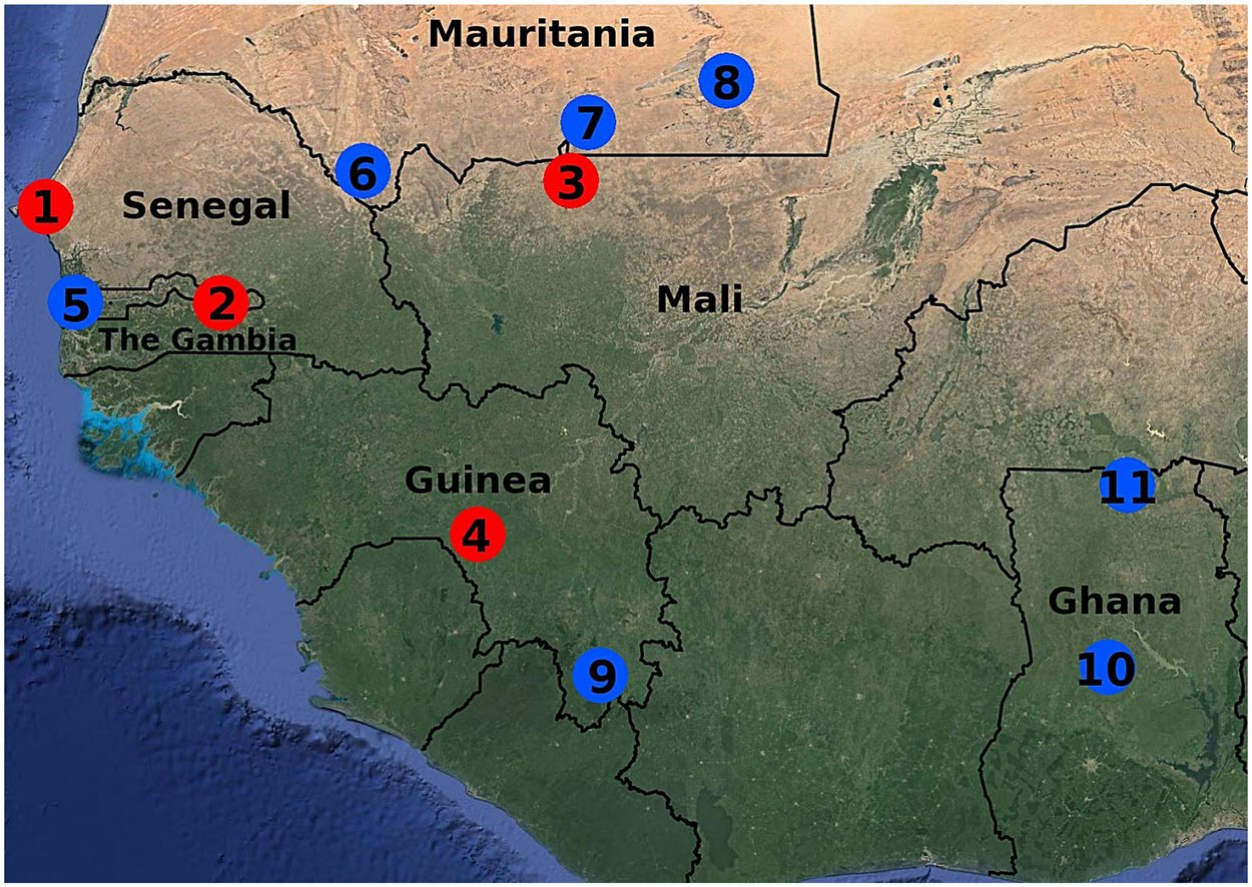
Locations of *Plasmodium falciparum* population samples from West Africa with genome sequences analysed in this study. Red points mark the location of the four newly sampled sites (1, Pikine in Senegal, 60 infections sequenced; 2, Basse in The Gambia, 113 infections sequenced; 3, Nioro in Mali, 52 infections sequenced; 4, Faranah in Guinea, 59 infections sequenced), and blue points indicate locations of previous population samples with which these were compared (5, Greater Banjul in The Gambia; 6, Selibaby in Mauritania; 7, Kobenni in Mauritania; 8, Nema in Mauritania; 9, N′Zerekore in Guinea; 10, Kintampo in Ghana, 11, Navrongo in Ghana). Further details are given in Table 1. Satellite image view shows diverse levels of vegetation cover due to varying amounts of annual rainfall across the region, reproduced with permission from Google Maps (Map Data ©2017 Google; Imagery ©2017 TerraMetrics; https://www.google.com/permissions/geoguidelines.html).

*P. falciparum* genome sequence data were obtained for 284 clinical malaria infections from newly sampled endemic sites in four West African countries (Fig. 1, Table 1 and Supplementary Table S1). Combined with data from other populations for which we had previously obtained sequences of at least 20 infection samples per site^8–10,20^, this yielded an overall genome sequence dataset for 680 infection samples from 11 sites in six countries across West Africa (Table 1). Filtering for highest sequence quality and read depth coverage yielded a set of 214 of the new samples and 319 of the previous samples in which a total of 365,876 high quality SNPs were reliably genotyped with <5% missing sample data per SNP, and <5% missing SNP data for any sample. The majority allele read for each SNP within each isolate was scored in order to count allele frequencies at each geographical site. Although many SNPs represented very rare variants, consistent with previous findings of an excess of rare variants in Africa^21^, 135,708 (37.1%) were non-singletons, and 35,228 (9.6%) had minor allele frequencies of greater than 1% in the overall dataset.

**Table 1:** Sequenced population samples of *Plasmodium falciparum* from four West African locations and previous data incorporated into a multi-population analysis across the region.

Country	Location	Year of sample collection	Number of infections sequenced (number in analysis)	Mean *F*_WS_^a^	Genome sequence data^b^
*New population samples*					
Mali	Nioro	2014	52 (51)	0.92	Table S1
Senegal	Pikine	2014	60 (59)	0.95	Table S1
The Gambia	Basse	2014	113 (80)	0.90	Table S1
Guinea	Faranah	2013	59 (24)	0.83	Table S1
*Previous population samples*					
The Gambia	Greater Banjul	2008	79 (64)	0.93	ref.^6,20^
Guinea	N’Zerekore	2011	134 (109)	0.84	ref.^10,21^
Ghana	Kintampo	2011	58 (44)	0.84	ref.^10,21^
Ghana	Navrongo	2011	48 (37)	0.84	ref.^8,21^
Mauritania	Kobenni	2014	23 (19)	0.96	ref.^9^
Mauritania	Nema	2014	33 (20)	0.87	ref.^9^
Mauritania	Selibaby	2014	21 (18)	0.88	ref.^9^

^a^*F*_WS_ values (within-infection genotypic fixation indices) are given for individual infection samples in Table S1 and Fig. 2.

^b^Sequence accession numbers for all 284 new *P. falciparum* clinical samples obtained from four populations in this study are given in Table S1. Sequence data from other populations are previously published, and SNP genotype data are publicly available https://www.malariagen.net/projects/p-falciparum-community-project. For published population data, sample sizes analysed here differ slightly from those previously analysed, due to improvements in the SNP calling algorithm, and also to criteria for exclusion based on missing data as described in the Materials and Methods.

### Within-infection sequence diversity in each local population

**Figure 2:**
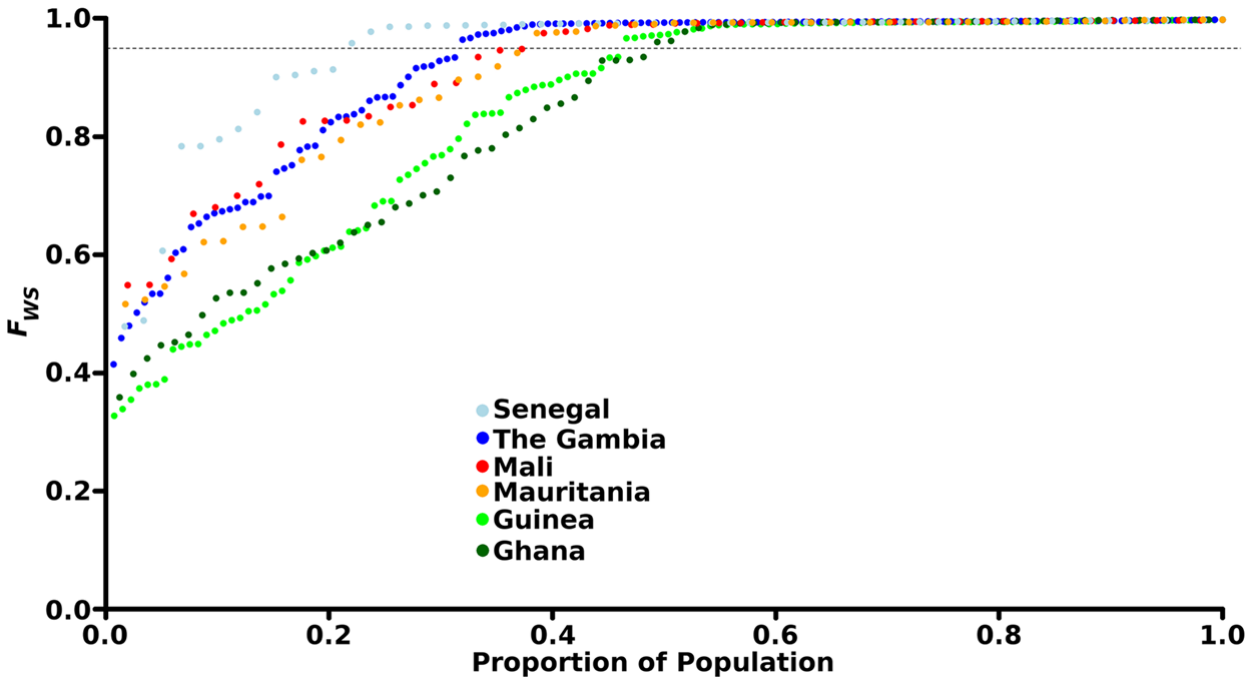
Analysis of genomic diversity within all *P. falciparum* infection samples analysed from six West African countries using genome wide sequence data. The fixation index *F*_WS_ is an inverse measure of within-infection diversity compared to the overall diversity in the population, so that a value of 1.0 indicates no variation within an infection, and lower values indicate infections with more diversity. Each point plots the value of an individual infection sample, and for each country the values are plotted in ascending order so that the cumulative proportions may be read along the x-axis. The dashed line corresponds to *F*_WS_ = 0.95, an arbitrary cut-off measurement above which an infection is considered to be virtually dominated by a single genotype. Mean values for each sampled site are shown in Table 1, and values for all individual infections are given in Table S1.

The extent of sequence diversity within each infection sample compared to the local population diversity was assessed using the *F*_WS_ fixation index, which has a potential range from zero (where an infection contains as much diversity as the total seen across the local population) to 1.0 (an infection containing no detectable sequence heterogeneity). The mean *F*_WS_ of infections at the four new sampled sites ranged from 0.83 (for the site at Faranah in Guinea where malaria is highly endemic) through to 0.95 (for the site with low endemicity at Pikine in Senegal), with the values for Basse in The Gambia and Nioro in Mali being intermediate (Table 1). This trend was as expected, and the values overlapped with the range seen at previously sampled sites in West Africa (Table 1 and Fig. 2). Comparing data across the six countries, the proportions of infections dominated by single genotypes (having *F*_WS_ indices >0.95; if such infections contained any unrelated mixed genotypes they could only be a very small proportion of parasites in any infection) ranged from 52% in Ghana to 80% in Senegal (Fig. 2). In pairwise comparisons of all countries, there was a significantly higher proportion of single genotype-dominated infections in Senegal than in either Mauritania, Guinea and or Ghana (Fisher’s exact tests, P < 0.05), and a significantly higher proportion of single genotype-dominated infections in The Gambia than in Guinea or Ghana (Fisher’s exact tests, P < 0.05).

### Signatures of haplotype homozygosity indicating recent directional selection

For each of the four newly sampled endemic locations, we examined the patterns of breakdown of haplotype homozygosity using the iHS index (standardised integrated haplotype score), calculated for all SNPs with a local minor allele frequency of >5% (Supplementary Fig. S1 and Supplementary Datafile S1). The iHS index is derived by comparing apparent haplotype breakdown between different alleles at each locus, so is not highly affected by differences in overall recombination rate or its estimation in different population samples^22^. All samples were included, rather than only single genotype infections, as analysis of previously studied populations yielded similar results with either approach^10^. Results revealed a number of genomic loci at which there were clearly high iHS values in multiple populations (Supplementary Fig. S1), including strong signatures around the chloroquine resistance transporter *crt* (on chromosome 7) and antifolate drug resistance gene *dhfr* (on chromosome 4), as seen in previously analysed populations and consistent with the history of drug treatment in this region^8–10,13,23^. A strong iHS signature near the end of chromosome 6 was also detected, as previously observed in other populations^8–10,13,23^, for which the cause of selection is unknown.

**Figure 3:**
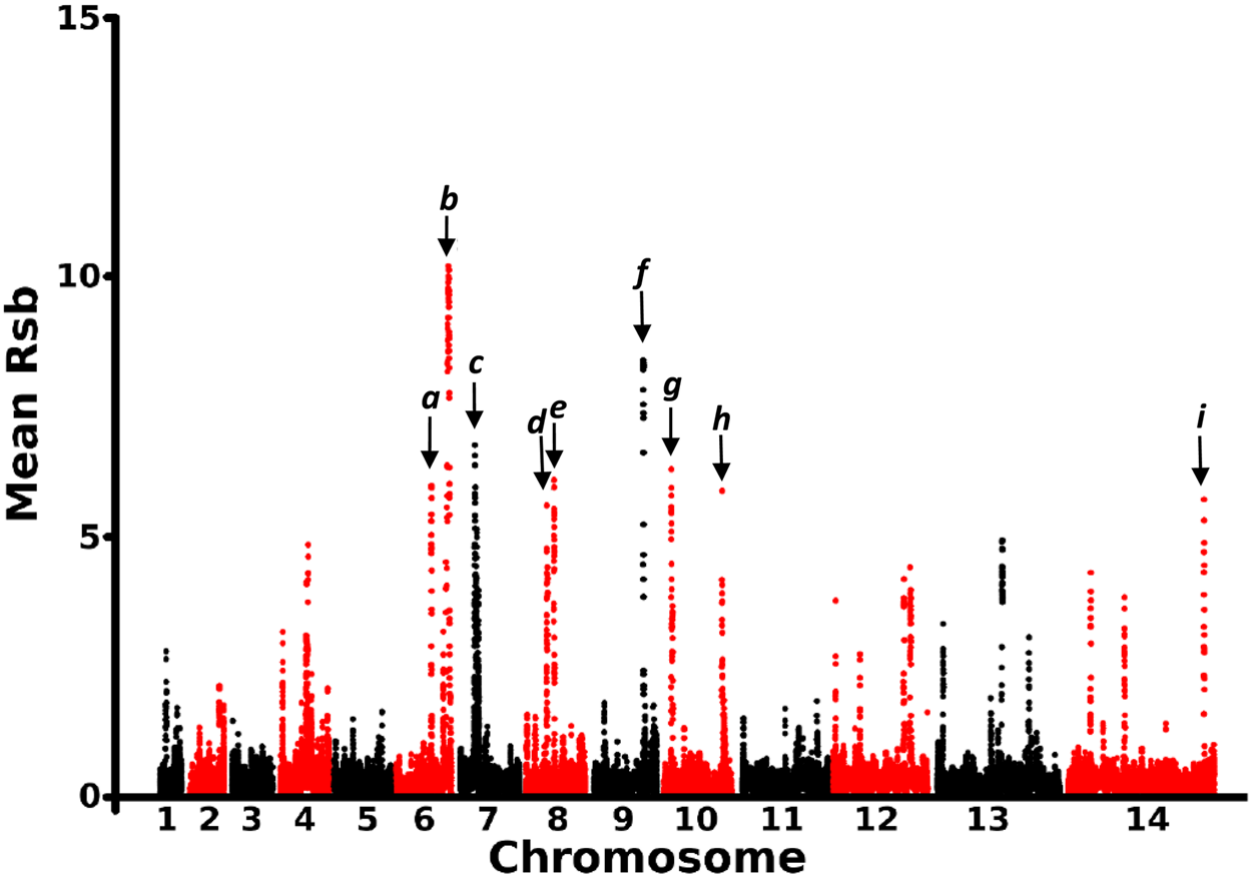
Mean Rsb index scores of SNPs across the *P. falciparum* genome over all pairwise comparisons of 11 local populations in West Africa. This index of the relative rate of breakdown of haplotype homozygosity is used here to identify loci with the most substantial differences in extended haplotypes among the populations, as this may indicate local differences in recent selection. All population pairwise Rsb values were determined for 35,228 SNPs, and the mean score for all pairwise comparisons was calculated from the absolute score magnitudes (Supplementary Datafile S2). The top nine genomic windows each had at least three SNPS with a mean Rsb > 3 and at least one SNP with a mean Rsb > 5: (*a*) chromosome (chr) 6 coordinates 852–860 kb covering 4 genes (PF3D7_0620400-0620700), (*b*) chr 6 coordinates 1174–1253 kb covering 17 genes (PF3D7_0628400-0630000 including the merozoite surface protein gene *msp10*), (*c*) chr 7 coordinates 385–467 kb covering 19 genes (PF3D7_0708500-0710200 including the chloroquine resistance gene *crt*), (*d*) chr 8 coordinates 523–549 kb covering 7 genes (PF3D7_0810200-0810800 including the antifolate resistance gene *dhps*), (*e*) chr 8 coordinates 678–691 kb covering 5 genes (PF3D7_0813900-0814300), (*f*) chr 9 coordinates 1167–1176 kb covering 3 genes (PF3D7_0929200-0929400 including the merozoite invasion-related gene *RhopH2*), (*g*) chr 10 coordinates 237–269 kb covering 10 genes (PF3D7_0929200-0929400), (*h*) chr 10 coordinates 1352–1360 kb covering 3 genes (PF3D7_0929200-0929400), (*i*) chr 14 coordinates 3018–3027 kb covering 3 genes (PF3D7_1474000-1474200).

To scan for evidence of differences in recent selection between all of the sampled endemic sites in the region, the relative breakdown in haplotype homozygosity with distance from each SNP was compared between populations using the Rsb metric (which contrasts data for each pair of populations sampled). Values were calculated for all pairs of endemic sites, excluding Selibaby in Mauritania due to a high frequency of identical genotypes at this site^9^. For all pairs of endemic sites, the Rsb values were determined for all 35,228 SNPs with overall minor allele frequencies of above 1% (Supplementary Datafile S2), and the mean of absolute values across all pairs was analysed. This identified nine genomic windows with elevated mean Rsb scores indicative of differences in the strength or timing of selection among populations, each window containing at least three SNPs having mean Rsb > 3 and at least one SNP with mean Rsb > 5, (Fig. 3 and Table 1). The strongest signature was located near the end of chromosome 6, mapping to the region noted above as having a signature of selection in individual populations indicated by iHS scores (Supplementary Fig. S1)^8–10,13,23^, with unusually long-range haplotypes^24^. Strong Rsb signatures were also observed around the drug resistance genes *crt* on chromosome 7 and *dhps* on chromosome 8, indicating the impact of historical variation in drug selection between different local populations. Other windows contain genes for which mechanisms of selection are unknown, including two that encode proteins of the merozoite stage which invades erythrocytes. The merozoite surface protein 10 (encoded by *msp10* in the first window on chromosome 6) is potentially subject to selection by acquired immune responses^25^, and a merozoite rhoptry protein (encoded by *RhopH2* in the window on chromosome 9) may be a target of immunity or local selection on parasite growth due to its additional role in nutrient uptake through the infected host erythrocyte membrane^26^.

### Genome-wide analysis of allele frequency distributions

**Figure 4:**
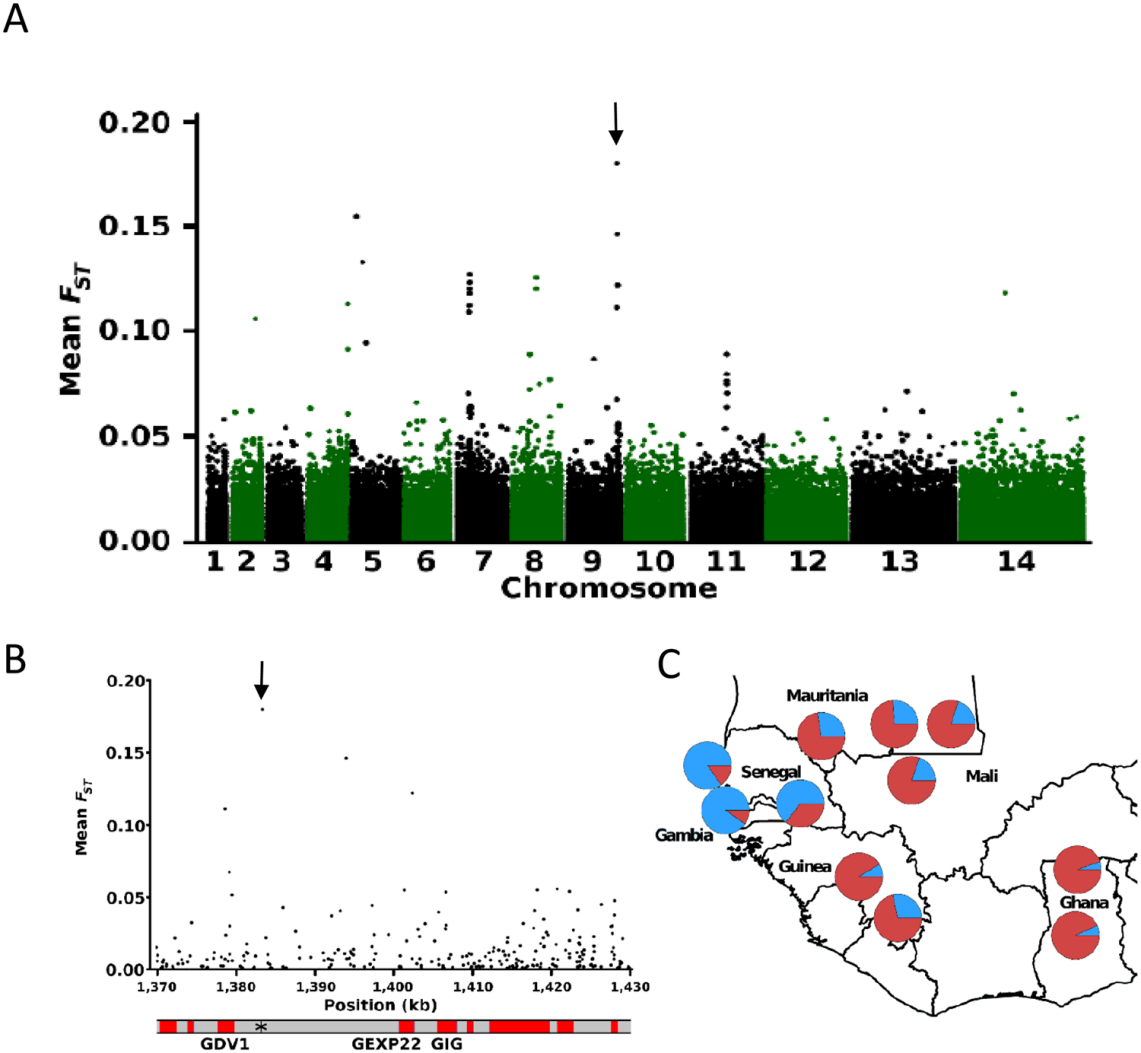
Scan for allele frequency divergence among 11 populations sampled across West Africa. (**A**) Mean *F*_ST_ scores for all pairwise site by site comparisons plotted for each SNP across the genome (the overall genome wide mean *F*_ST_ was 0.007). The arrow shows the locus with highest mean *F*_ST_ value (*F*_ST_ = 0.18 for an intergenic SNP at position 1,383,344 of chromosome 9 adjacent to the *gdv1* gene PF3D7_09035400). Table 2 lists this and three other genomic regions that each contained at least three SNPs with mean *F*_ST_ > 0.05 and at least one SNP with mean *F*_*ST*_ > 0.1 (with <15 kb between adjacent SNPs having these high values). (**B**) Zoomed in view of *F*_ST_ values of the SNPs in the most highly differentiated region on chromosome 9, with the highest *F*_ST_ SNPs spanning the region between *gdv1* and *gexp22*. (**C**) Geographical allele frequency distribution of chromosome 9 SNP at position 1383344, with pie charts showing the reference 3D7-type allele (red), and the alternate allele (blue). Allele frequency distributions for the highest *F*_ST_ SNP in each of the other three high *F*_ST_ windows are shown in Supplementary Fig. S2.

To test for geographical divergence in allele frequencies of individual SNPs across the genome, pairwise *F*_ST_ values were calculated for all non-singleton SNPs for each combination of sample sites. The overall genome wide divergence averaged across all population pairs was very low (*F*_ST_ = 0.007), with only 17 SNPs each having overall mean pairwise *F*_ST_ values of >0.10 (Fig. 4A). There were four discrete genomic windows containing multiple SNPs with elevated *F*_ST_ values (Table 2). The SNP with the genome-wide highest *F*_ST_ was on chromosome 9, within a window containing four SNPs having *F*_ST_ values > 0.1 spanning the *gdv1* gene (Pf3D7_0935400) and its 3′-intergenic region up to the adjacent *gexp22* gene (Pf3D7_0935500) (Fig. 4B and Table 2). In addition to evidence that *gdv1* is critical to early gametocyte development^16^, it is notable that the product of *gexp22* is specifically exported in early gametocytes^27^, and genetic manipulation of the next adjacent coding sequence (*gig*, ‘gametocytogenesis implicated gene’) has been shown to affect levels of gametocyte production in culture^28^. Analysis of the allele frequency distribution of the highest *F*_ST_ SNP (position 1,383,344 on chromosome 9) showed that the non-reference allele had a high frequency only in the three populations sampled in The Gambia and Senegal (Fig. 4C). The other separate genomic windows with high *F*_ST_ included a *hyp15* gene on chromosome 4, the chloroquine resistance transporter *crt* gene on chromosome 7, and a gene of unknown function on chromosome 8, each of which showed geographical patterns that did not match that of the top *F*_ST_ SNP on chromosome 9 (Supplementary Fig. S2).

**Table 2:** Genomic windows of *Plasmodium falciparum* with highest geographical differentiation across the full set of West African populations analysed.

Chromosome	position on chromosome in kb	Number of SNPs	Highest mean *F*_ST_	Number of genes	Gene loci^*^
4	1137–1142	3	0.113	2	PF3D7_0425250-0425300
7	371–435	14	0.127	20	PF3D7_0708000-0709700
8	701–703	3	0.126	1	PF3D7_0814500
9	1378–1422	11	0.180	6	PF3D7_0935400-0935900

^*^Each window contains at least 3 SNPs with mean *F*_ST_ (across all population pairs) > 0.05 and at least one SNP with mean *F*_ST_ > 0.10. Co-ordinates and gene loci are as described in *the P. falciparum* 3D7 reference genome version 3.1, which may be visualised in detail through the PlasmoDB genome browser^35^.

### Mutually exclusive allelic deletions in the intragenic region between gdv1 and gexp22

**Figure 5:**
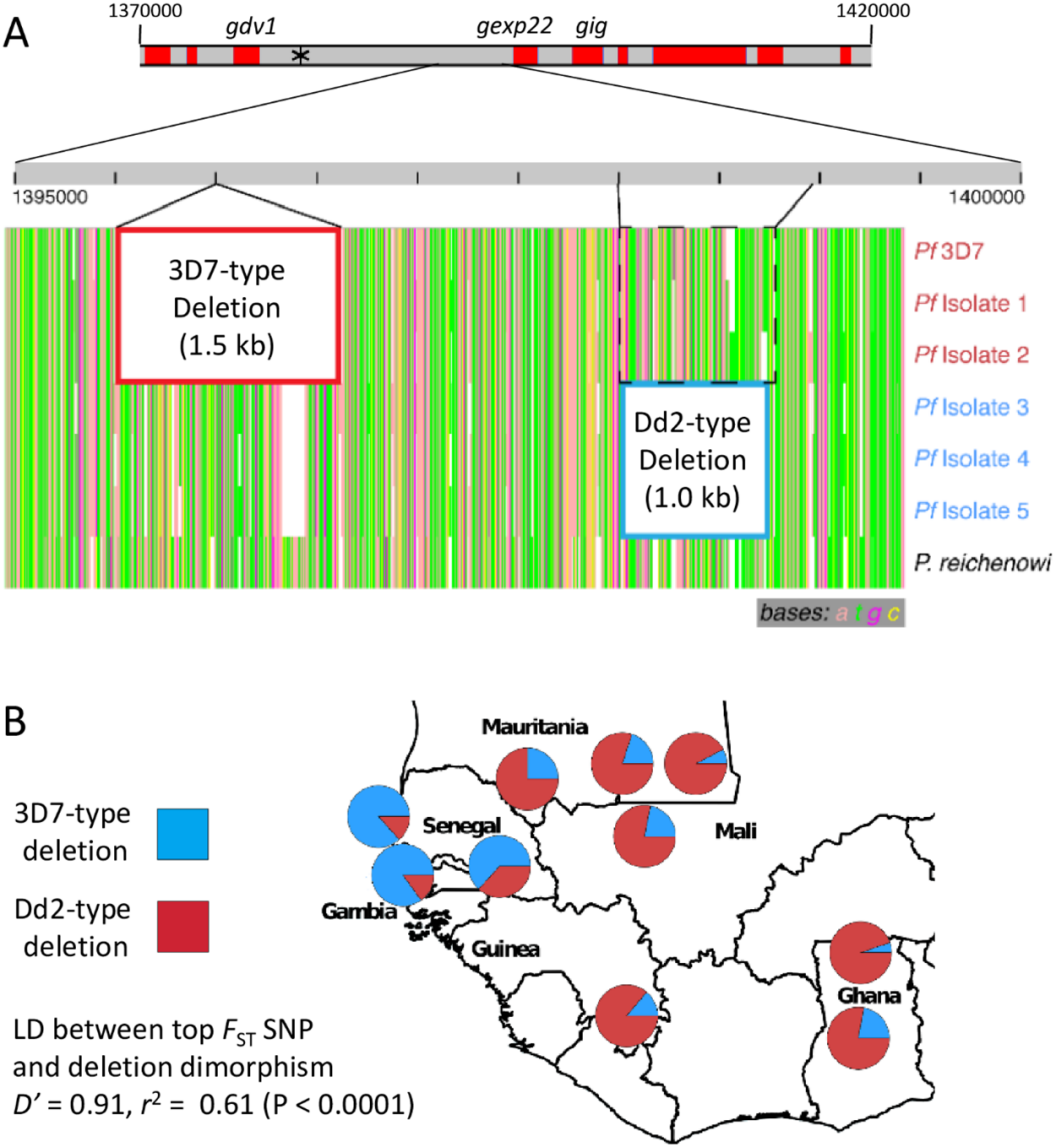
Mutually exclusive large allelic deletions on chromosome 9 within the high *F*_ST_ window between *gdv1* and *gexp22*. (**A**) Map of a 60 kb region of the chromosome (from position 1,370,000–1,430,000) marking the individual genes, and zoom into a 5 kb part of the intragenic region of the reference 3D7 sequence. The position of the highest *F*_ST_ SNP is shown with an asterisk. The PacBio long-read sequence pileup of the region from six *P. falciparum* isolates shows the two allelic deletion blocks, termed the 3D7 type (1.5 kb deletion outlined in red) and Dd2 type (1 kb deletion in blue). PacBio sequencing of the closely related species *P. reichenowi* identified the presence of both insert blocks, indicating the deletion event occurred subsequent to the separation of these two species. (**B**) Map showing pie charts of the frequencies of the alternative allelic deletion alleles across West Africa, 3D7 reference type (red) and Dd2 type (blue). Short read data from single genotype infections were mapped to a partial chromosome 9 reference containing both the 3D7 and Dd2 insert types, allowing proportions of alleles to be determined at all except one of the local populations (Faranah in Guinea for which mapped read depths were too low for most isolates). The deletion alleles were in strong overall linkage disequilibrium (LD) with the alleles at the highest *F*_ST_ SNP (*D*′ = 0.91, *r*^2^ = 0.61), a similar association seen within most of the local populations analysed separately (mean *D*′ = 0.93, mean *r*^2^ = 0.53).

Analysing all samples together shows an unusually high level of linkage disequilibrium among SNPs in the chromosome 9 region adjacent to *gdv1* (Supplementary Fig. S3), contrasting with very low levels of disequilibrium in most of the *P. falciparum* genome in all West African populations sampled here or previously^9,10,20,23^. This region on chromosome 9 has undergone large chromosomal deletions in some long-term laboratory adapted parasite isolates^29,30^, and apparently in a few clinical isolates after several months of culture^31^. Deletions have been associated with a loss of sexual stage gametocytogenesis among laboratory lines^30^, and thereby inability for parasites to be transmitted to mosquitoes. To examine whether naturally occurring deletion polymorphisms may be present, long-read PacBio sequence data for several isolates were examined, including new clinical isolates (Fig. 5A). Comparison of these sequences alongside that from a closely related chimpanzee parasite species *P. reichenowi* identified including a previously undescribed major allelic dimorphism in the 3′-intergenic sequence of *gdv1* in *P. falciparum* (Fig. 5B). The first allelic sequence segment is 1.5 kb in length and absent in the *P. falciparum* 3D7 reference strain, while the second allelic segment is present in 3D7 and corresponds to a 1 kb sequence beginning 2 kb downstream along the chromosome towards the adjacent *gexp22* gene (Figs 5B and S4). These data from a small number of isolates allow definition of two divergent *P. falciparum* alleles as ‘3D7-type’ (lacking segment 1 while possessing segment 2), and the alternative ‘Dd2-type’ (possessing segment 1 but missing segment 2), the segments appearing mutually exclusive in *P. falciparum* alleles, whereas both are present in *P. reichenowi*.

To genotype these allelic deletions in additional samples of *P. falciparum*, PCR protocols were developed to amplify both segments, which confirmed that they are mutually exclusive among 13 culture adapted *P. falciparum* laboratory lines of diverse geographical origins (Supplementary Table S2). Each parasite line had either the 3D7-type (lacking segment 1 but possessing segment 2), or the alternative (possessing segment 1 but lacking segment 2) which is here termed the Dd2-type. The dimorphic allelic products were of the exact expected sizes in all of the respective cultured parasites, indicating that there is only one deletion allele of each type.

To determine the frequency of the dimorphic deletion genotypes across West Africa we remapped the short read Illumina data from each of the populations to a hybrid chromosome 9 sequence possessing both of the sequences of the deleted segments, and determined the sequence coverage against each allelic segment. All samples considered to contain predominantly single clone infections (*F*_WS_ values of >0.95) were determined to contain either the 3D7-type or Dd2-type deletions, and no genotypes with neither or both deletions were seen. The geographical distribution of the alternative deletion alleles (Fig. 4C) was closely correlated with the allele frequencies seen for the highest *F*_ST_ SNP (at chromosome 9 position 1,383,344), with which it was in very strong linkage disequilibrium overall (*D*′ = 0.91, *r*^2^ = 0.61; P < 0.0001). This linkage disequilibrium was similar within most of the local populations analysed separately (mean *D*′ = 0.93, mean *r*^2^ = 0.53). Each of the populations sampled in The Gambia and Senegal had the Dd2-type deletion allele at high frequency, whereas this was at low frequency in each of the other populations in West Africa. The highest frequencies of 87% and 83% were at the coastal sites of Pikine in Senegal and Greater Banjul in The Gambia, respectively. Short read sequences available from samples of other geographical sites (https://www.malariagen.net/projects/pf3k) indicate that the Dd2-type deletion allele is common in Southeast Asia but generally uncommon in Africa, confirming that the high frequencies identified here in The Gambia and Senegal are exceptional within the region.

To assess whether the local frequency of the deletion alleles has changed over time in an area with exceptional frequency, samples previously collected in 2001 from the Greater Banjul area in The Gambia were genotyped by PCR. This revealed that the Dd2-type deletion allele had a high frequency (72%, the major allele within 44 of 61 clinical infection samples genotyped), similar to that in the more recent samples from the same area.

### Assay of parasite gene transcript levels in clinical malaria samples using RT-qPCR assays

**Figure 6:**
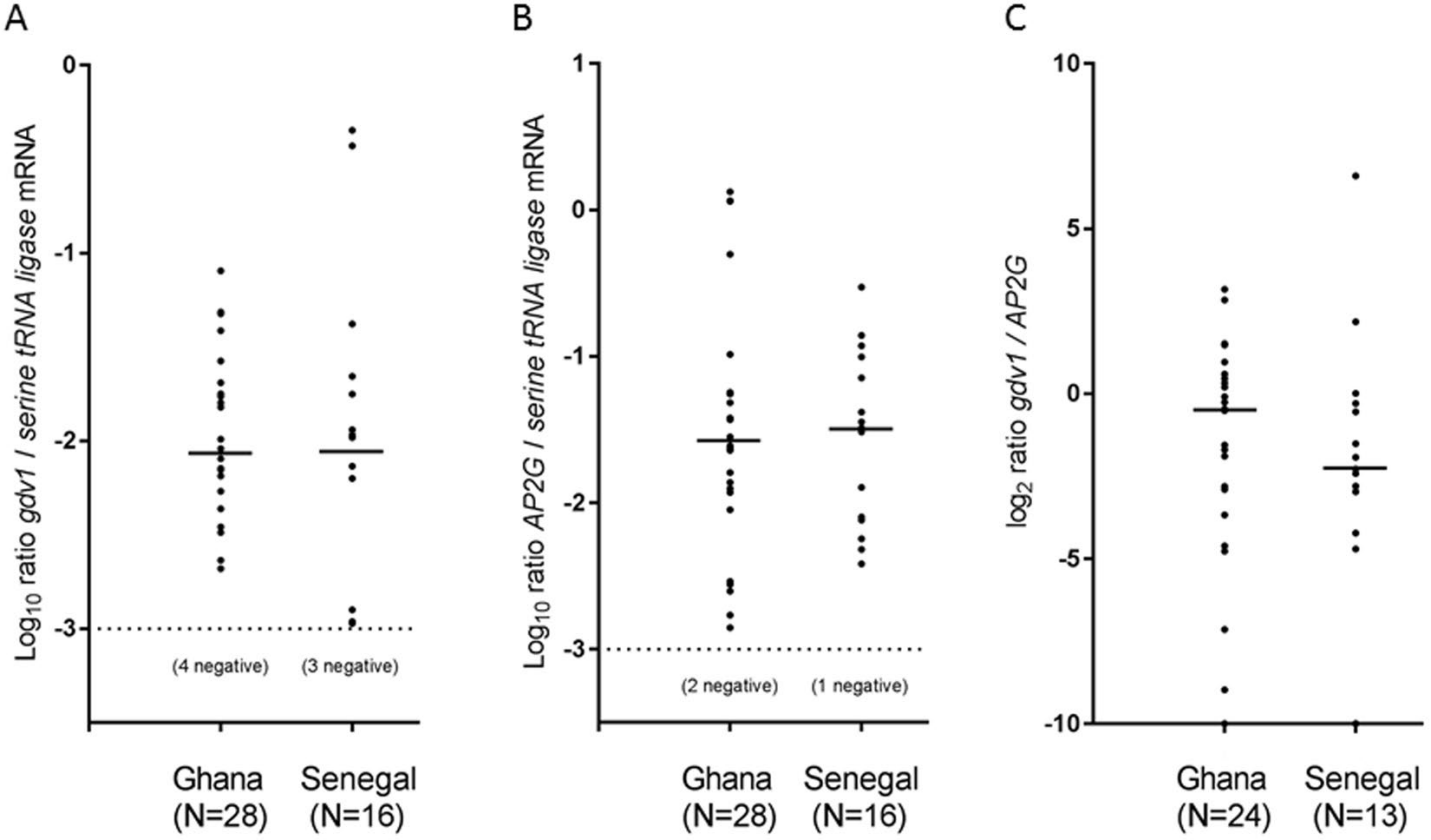
Estimation of *gdv1* and *AP2G* transcripts in 44 individual *P. falciparum* clinical infection samples from Ghana and Senegal as measured by RT-qPCR using primer pairs within each coding sequence. (**A**) Ratios (Log_10_) of *gdv1* to a housekeeping gene (*serine tRNA ligase*) show a wide spectrum that is similar in each population. (**B**) Ratios (Log_10_) of *AP2G* to the housekeeping gene shows a similarly wide spectrum that does not differ between the populations. (**C**) Ratios (Log_2_) of the two gametocyte commitment genes *gdv1* / *AP2G* (these ratios were not determined for 7 of the clinical infections due to undetectable transcript measurement for either gene). These distributions are not significantly different between the two countries (Mann-Whitney P > 0.1). It should be noted that the assays do not discriminate between sense mRNA and antisense transcripts which have recently been described to be involved in regulation of *gdv1*^50^.

Levels of mRNA transcripts for *gdv1*, and the gene encoding transcription factor AP2G which is required for enabling early stage sexual development in laboratory culture-adapted parasites^15,32^, were explored in clinical samples. Transcript levels of the *AP2* gene in clinical isolates have been described to vary among different endemic areas of East Africa^33^, but the timing of expression of that gene during synchronised parasite culture appears to differ from that of *gdv1*^34^. Both of these genes, as well as a gene highly expressed in mature late stage gametocytes (*Pfs25*) and a housekeeping gene (*serine tRNA ligase*) were assayed by RT-qPCR of RNA extracted from blood samples of 81 clinical malaria patients in two populations with contrasting infection endemicity (Kintampo in Ghana and Pikine in Senegal). The RT-qPCR assay data for 44 isolates (28 from Ghana and 16 from Senegal) had >1000 copies per microlitre measured for the housekeeping control gene, and these were selected as most reliable for quantitative analysis. The levels of *gdv1* and *AP2G* transcripts detected relative to the housekeeping gene showed a very wide range among the individual clinical infections, but did not differ between the two endemic populations (Fig. 6A,B; Mann-Whitney P > 0.10 for each comparison). The transcript levels of neither gene correlated significantly with those of the late stage gametocyte *Pfs25* gene (r = 0.09 for *AP2G* vs *Pfs25*, r = 0.07 for *gdv1* vs *Pfs25*, P > 0.5 for each). In relation to each other, the ratio of *gdv1* to *AP2G* transcripts measured in each isolate showed a broad range, with a median ratio in Ghana (0.722) that was three times higher than that measured in Senegal (0.234), although there was a largely overlapping distribution in both populations (Fig. 6C; Mann-Whitney P > 0.10).

## Discussion

To identify parasite genomic loci under divergent selection among local endemic populations, this study focused on multiple different sites in West Africa where selection would operate against a background of extensive gene flow that keeps allele frequencies similar among populations throughout most of the genome. Aside from confirming significant differential selection on drug resistance genes for which varying local selection history is already known, the analysis has revealed several loci at which there have been local differences in selection, for which the causes are not yet known. Overall, the locus with most exceptional differentiation in a genome-wide scan is the gametocyte development gene *gdv1* and its 3′-intergenic region, the largest intragenic region of the *P. falciparum* genome^35^ which is here shown to contain a remarkable structural dimorphism.

Although SNP data are technically most robust for population genomic analyses of *P. falciparum*, it is now known that insertions, deletions and other structural variations account for a considerable part of the genomic diversity among laboratory parasite lines^36^. Copy number variation (CNV) among clinical isolates has been surveyed using microarrays^31,37^, and some CNV loci can be genotyped using paired-end short-read sequence data, but characterisation of complex structural polymorphism may be most clearly resolved with long-read sequencing. Here, the identification of two major allelic deletions in the *gdv1* 3′-intergenic region involved focused inspection of long-read sequence data for a small number of *P. falciparum* isolates, which then led to analysis of a large numbers of samples, firstly by mapping of short-read sequences to the alternative allelic reference sequences, and secondly by design of primers for PCR-based genotyping. This confirmed the complete allelic exclusivity of each deletion and enabled their local frequencies to be determined. Remarkably, the Dd2-type allele of the *gdv1* 3′-intergenic sequence occurs at very high frequencies in the coastal populations in The Gambia and Senegal in the extreme west of Africa, but is relatively rare elsewhere in the region. In a laboratory parasite line the locus shows distinctive long non-coding RNA transcripts, one of which includes the entire antisense of the *gdv1* coding sequence and another spans the intergenic region with dimorphic deletions, suggesting that a complex transcriptional regulatory process may control the onset of parasite sexual development^18,19^.

Population differentiation at the *gdv1* locus may be affected by varying levels of malaria infection endemicity or different vector population ecology. It is notable that malaria incidence has declined in The Gambia and Senegal more markedly than reported elsewhere in West Africa to date^38–40^. However, retrospective analysis of samples from The Gambia show that the allele frequency has not changed significantly since 2001, despite a major local decline in malaria incidence after this time^39,41^. It is also the case that populations surveyed in Mauritania do not have significantly different allele frequencies from other sites in the region, although *P. falciparum* endemicity in that country is lower than in other areas apart from Senegal and The Gambia^9^. Therefore, selection may be due to subtle differences in transmission ecology rather than crude differences in levels of infection locally.

It is clear that mosquito vectors of malaria are locally variable across West Africa, with different members of the *Anopheles gambiae* complex, as well as members of the *An. funestus* complex and other species contributing to transmission^42–44^. In The Gambia and coastal areas of Senegal, where tidal effects cause brackish waters to extend inland in riverine systems, *An. melas* is a dominant member of the *An. gambiae* complex^45^. Moreover, The Gambia and Senegal are part of a unique zone in the extreme western part of Africa where there is extensive hybridisation between the recently separated sibling species, *An. gambiae* sensu stricto and *An. coluzzii*^45–47^. Furthermore, additional differences in population structure within both *An. gambiae* and *An coluzzii* between coastal and inland populations in The Gambia and Senegal indicate that *P. falciparum* is transmitted by significantly different vector populations that are locally adapted^48^. Differences in vector populations might select for population differentiation at the *gdv1* locus, and surveys to analyse parasites directly within the vectors should be undertaken, although these will be laborious due to low proportions of mosquitoes being infected. Interestingly, a recent genome-wide survey of CNV variation at three sites in East Africa showed a generally very rare deletion removing *gdv1* and distal genes at the end of chromosome 9 to be at appreciable frequency in samples from a site in Sudan where there is low seasonal transmission^49^. Although that study was not designed to detect common polymorphism including the intergenic dimorphism described here, it suggests that differences in transmission elsewhere might cause local selection on rare variants.

There is a need to develop robust assays of parasite function and gene expression at the early sexual stage of development in *P. falciparum*^15^. Transcript analysis by RT-qPCR here showed that in comparison with a housekeeping gene there was variation among different infections in the relative levels of *gdv1* transcripts, and in levels of transcripts of the *AP2G* gene separately implicated in gametocyte differentiation, as expected given that levels of gametocytes vary widely between individual infections. Although it was recently suggested that *AP2G* transcription may alter in response to different environments^33^, the distributions of relative transcript levels were not significantly different between two populations with respectively low and high endemicity here. It should be noted that these RT-qPCR assays based on double-stranded cDNA do not discriminate between sense and antisense transcripts, which are a feature of the *gdv1* locus^18,19,50^. It will be important for future studies to obtain strand-specific RNAseq data from clinical samples, to test whether the polymorphism directly affects non-coding RNA-mediated regulatory mechanisms that control *gdv1* gene function. This is a particularly exciting prospect in the light of a key study that has just shown the role of antisense RNA in silencing the transcription of *gdv1* mRNA and repressing commitment to sexual stage development in laboratory culture^50^. As many *P. falciparum* lines cultured for a long time contain loss-of-function mutants^51^, some of which are associated with loss of ability to make gametocytes^30,32^, functional analysis of the natural polymorphism should be performed on parasite isolates cultured from relevant endemic populations such as those identified here.

## Materials and Methods

### Study sites, sample collection and preparation of DNA for genome sequencing

Clinical malaria samples that were *P. falciparum* positive by slide microscopy were collected from patients presenting at local health facilities at four geographic locations, in Mali (Nioro du Sahel: Lat 15.183, Long -9.550), Senegal (Pikine: Lat 14.765, Long -17.391), The Gambia (Basse: Lat 13.317, Long -14.217), and Guinea (Faranah: Lat 10.040, Long -10.743) (Fig. 1 and Table 1). Clinical malaria infections were diagnosed using rapid diagnostic tests or blood film inspection, and venous blood samples of up to 5 ml were collected from consenting patients into anticoagulant-containing tubes. Approval to collect and analyse the clinical samples for this study was granted after review by the Joint Ethics Committee of the Gambian Government and the MRC Gambia Unit, the Ethics Committee of the Ministry of Health in Senegal, the Ethics Committee of the Ministry of Health in Mali, the Ethics Committee of the Ghana Health Service, the Ethics Committee of the Noguchi Memorial Institute for Medical Research, the University of Ghana Ethics Committee, the Ethics Committee of the Kintampo Health Research Centre, the Ethics Committee of the Navrongo Health Research Centre, the Ethics Committee of the Ministry of Health in Mauritania, and the Ethics Committee of the London School of Hygiene and Tropical Medicine. Written informed consent was obtained directly from adult subjects and from parents or other legal guardians of all participating children, and additional assent was received from children themselves if they were 10 years of age or older. All methods were performed in accordance with the relevant local and international guidelines and regulations.

Leukocytes were depleted from the blood samples using CF11 cellulose powder filtration columns, and the erythrocytes were frozen at −20 °C. DNA was extracted from the frozen blood containing parasites using the QIAamp blood Midi kit, and DNA samples of sufficient quantity and quality were processed for paired-end short read sequencing on an Illumina HiSeq, generating reads of up to 150 bp. These new data were analysed together with data we had previously generated from seven other sites, to allow comparisons among sites separated by up to 1800 km in this large contiguous region. The previous population data were from The Gambia (Greater Banjul area)^20^, Guinea (N′Zerekore)^10^, Ghana (Kintampo and Navrongo)^8^, and Mauritania (Selibaby, Kobenni and Nema)^9^, for which sequence data are publicly accessible and SNP genotypes have been made freely available through the MalariaGEN Plasmodium falciparum Community Project (https://www.malariagen.net/projects/p-falciparum-community-project).

### Sequence read mapping and SNP allele calling

Sequence reads for each of the 284 new *P. falciparum* infection samples have been deposited in the European Nucleotide Archive (accession numbers listed in Table S1). Reads and samples were quality controlled following the internal pipeline of the Wellcome Trust Sanger Institute prior to mapping to the *P. falciparum* 3D7 v3 reference genome. SNPs were called as part of the MalariaGEN pipeline version 6.0, and high quality SNPs were defined as those that were polymorphic within West Africa and which passed each of the VCF filters or which were only marked by the CodingType filter (allowing for the inclusion of intergenic SNPs). Short indel genotype calls were filtered from the dataset and not analysed here, whereas SNP genotype calls were made on each individual infection sample with a minimum per infection sample coverage at each SNP of 10 reads. Due to the presence of mixed clone infections SNP genotypes were called on a majority basis, the allele call with the highest number of supporting reads being defined as the majority allele in the infection. The population dataset was filtered in R using an iterative process to exclude samples and SNPs with excessive missing data. During the first iteration, samples with >90% missing data were removed prior to exclusion of SNPs with >90% missing data, and then the maximum allowed percentage of missing data was decreased in steps of 5% until all samples and SNPs had <5% missing data across the entire West African dataset.

### Genome-wide SNP data and population genetic analysis

The genotypic complexity of each infection was estimated using the within-infection diversity fixation index *F*_WS_ (with a potential range from zero to 1.0, the latter indicating no diversity within an infection). This was calculated using custom Perl and R scripts using total read depth data from the raw VCF files, and for this analysis a minimum total coverage of 20 reads at each SNP was required. Diversity within each infection sample was assessed relative to the total population, with a lower *F*_WS_ score indicating more diverse mixed genotype infections, and those with *F*_WS_ > 0.95 were considered as predominantly single genotype infections (within which any possible additional unrelated genotypes must be at low frequency).

An initial scan for evidence of recent directional selection in the four newly sampled populations was performed using the standardised integrated haplotype score (iHS). The iHS values were calculated for each SNP having a minor allele frequency of above 5% in each local population, using the REHH package for R^52^. Population specific recombination maps were independently estimated for each of the four populations using the LDhat program^53^, run with a block penalty of 5, 10 million rjMCMC iterations and a burn in of 100,000 iterations. For this analysis, the putative ancestral *P. falciparum* allele for each SNP was determined by alignment with the reference genome sequence of the closely related chimpanzee parasite *P. reichenowi* CDC-1 strain^54^, with SNP positions being discarded if an ancestral allele was undetermined. Population specific directional selection was assessed using the Rsb statistic^55^ for all possible population pairs with the exclusion of Selibaby in Mauritania (due to the high frequency of near identical genotypes within this population). Windows of population specific directional selection were identified by calculation of the mean |Rsb| score for each SNP in all pairwise comparisons of populations. For the Rsb analysis a single West African recombination map was estimated as the average of 5 independent runs of 100 samples randomly drawn from the total dataset. Allele frequency divergence between populations was summarised for all population pairs with the *F*_ST_ fixation index using custom R scripts. Outliers were identified based on the mean observed *F*_ST_, with high *F*_ST_ windows defined as containing 3 or more SNPs with mean *F*_ST_ > 0.05 and at least one SNP with mean *F*_ST_ > 0.1 (and <15 kb between adjacent SNPs having these values).

### Identification and genotyping of novel intergenic allelic deletions

For the intergenic sequence analysis, long read sequence data generated with the Pacific Biosciences SMRT sequencing platform data from a small panel of laboratory lines and recently adapted clinical samples in the Pf3K reference genomes project were examined (ftp://ftp.sanger.ac.uk/pub/project/pathogens/Plasmodium/falciparum/PF3K/PilotReferenceGenomes/). The reference sequence of *P. reichenowi* PrG01 which has been generated by long read sequencing using the Pacific Biosciences SMRT sequencing platform was also used for an outgroup comparison (http://biorxiv.org/content/early/2016/12/20/095679).

PCR based genotyping of the two major intergenic deletions between the *gdv1* and *gexp22* genes was performed on a panel of 13 cultured laboratory lines, and on archived samples of clinical infections collected in 2001 from the Greater Banjul area of The Gambia^56^. Primers were designed to genotype the presence of absence of each allelic large indel sequence using conserved flanking sequences in the 3D7 reference genome sequence and PacBio derived Dd2 sequence. The pair of primer sequences flanking the first indel (sequence segment present in Dd2 but not in 3D7) were Chr9-block1-F3 5′-acactgtttttgtaccgcattataaag-3′ and Chr9-block2-R2 5′-agcgtgtgtgtgaggaatgactc-3′, producing fragments of 739 bp in 3D7 and 2245 bp in Dd2. The pair of primer sequences flanking the second indel (sequence segment present in 3D7 but not in Dd2) were Chr9-block2-F1 5′-gtcttataagaagtcacagcttcatgt-3′ and Chr9-block3-R2 5′-cccagaaaaaggtaagtaagaaagt-3′, producing fragments of 2001 bp in 3D7 and 974 bp in Dd2. PCR amplifications were performed using TaKaRa high fidelity PCR Premix (Clontech, UK) in a volume of 5 ul, with 35 cycles of 95 °C for 30 seconds denaturation, 64.5 °C for 15 seconds annealing, and 70 °C for 3 minutes elongation, following which PCR products were separated on 1% agarose gels and stained with ethidium bromide for scoring of allelic product sizes.

### Analysis of gene transcript levels in clinical isolates

For RNA analysis by reverse transcription followed by quantitative polymerase chain reaction amplification (RT-qPCR), peripheral blood samples of 81 patients with clinical *P. falciparum* malaria infections were studied, 48 sampled at Pikine in Senegal, and 33 sampled at Kintampo in Ghana, with each sample containing at least 100 μL packed red blood cells cryopreserved within four hours of collection. Samples were shipped on dry ice (−78 ^o^C) to a single laboratory where they were thawed and TRIzol reagent (Invitrogen) was added before being stored at −80 °C. RNA extraction was performed using RNeasy Micro kits (Qiagen), and mRNA was reverse transcribed with oligo(dT) using TaqMan reverse transcription reagents (Life Technologies), to enable qPCR analysis of transcript levels of early markers of gametocyte commitment, *gdv1* (PF3D7_0935400) and *AP2-G* (PF3D7_1222600),) as well as a housekeeping gene serine tRNA ligase (PF3D7_0717700). Primer pairs for amplification of short target sequences within exons were as previously described for *gdv1* (5′-taggcgtcgaaatagtgctagtagaaa-3′ and 5′-gtcctcacaaccagcatcattagta-3′)^16^, for AP2G (5′-aacaacgttcattcaataaataagg-3′ and 5′-atgttaatgttcccaaacaaccg-3′)^34^, and for serine tRNA ligase (5′-aagtagcaggtcatcgtggtt-3′ and 5′-gttcggcacattcttccataa-3′)^16^. Reactions were conducted in 10 μL volumes using SYBR select master mix (Applied Biosystems) and 300 nM of each primer, on a Rotor-Gene 3000 qPCR machine at 50 °C for 2 minutes and 95 °C for 2 minutes, followed by 40 cycles at 95 °C for 15 seconds and 60 °C for 1 minute.

## Electronic supplementary material

Supplementary Figures and Tables

Dataset 1

Dataset 2

## Data Availability

All data are fully available without restriction. Paired-end short read genome sequence data for the 284 new parasite infection samples have been deposited in the European Nucleotide Archive, with accession numbers listed in Table S1. The short read sequences and SNP genotypes from previous population sample data are publicly available https://www.malariagen.net/projects/p-falciparum-community-project.
