# Supplementary material for: Multi-population genomic analysis of malaria parasites indicates local selection and differentiation at the gdv1 locus regulating sexual development
Source: Sci Rep. 2018 Oct 25;8:15763. doi: 10.1038/s41598-018-34078-3 (PMC6202401; doi:10.1038/s41598-018-34078-3)
Supplement: Supplementary file 1 — Supplementary Figures and Tables [file 41598_2018_34078_MOESM1_ESM.pdf]

Multi-population genomic analysis of malaria parasites indicates local selection and differentiation at the *gdv1* locus regulating sexual development

Supplementary Information:

Figures S1 - S4 and Tables S1 and S2

Craig W. Duffy[1], Alfred Amambua-Ngwa[2], Ambroise D. Ahouidi[3], Mahamadou Diakite[4], Gordon A. Awandare[5], Hampate Ba[6], Sarah J. Tarr[1], Lee Murray[1], Lindsay B. Stewart[1], Umberto D'Alessandro[2,8], Thomas D. Otto[7], Dominic P. Kwiatkowski[7], and David J. Conway[1*]

[1] Pathogen Molecular Biology Department, London School of Hygiene and Tropical Medicine, Keppel St, London, UK

[2] MRC Gambia Unit, Fajara, The Gambia

[3] Le Dantec Hospital, University Cheikh Anta Diop, Dakar, Senegal

[4] Malaria Research and Training Center, University of Bamako, Mali

[5] West African Centre for Cell Biology of Infectious Pathogens (WACCBIP) and Department of Biochemistry, Cell and Molecular Biology, University of Ghana, Legon, Ghana

[6] Institut National de Recherches en Santé Publique (INRSP), Nouakchott, Mauritania

[7] Malaria Programme, Wellcome Trust Sanger Institute, Cambridge, UK

[8] Disease Control Department, London School of Hygiene and Tropical Medicine, Keppel St, London, UK

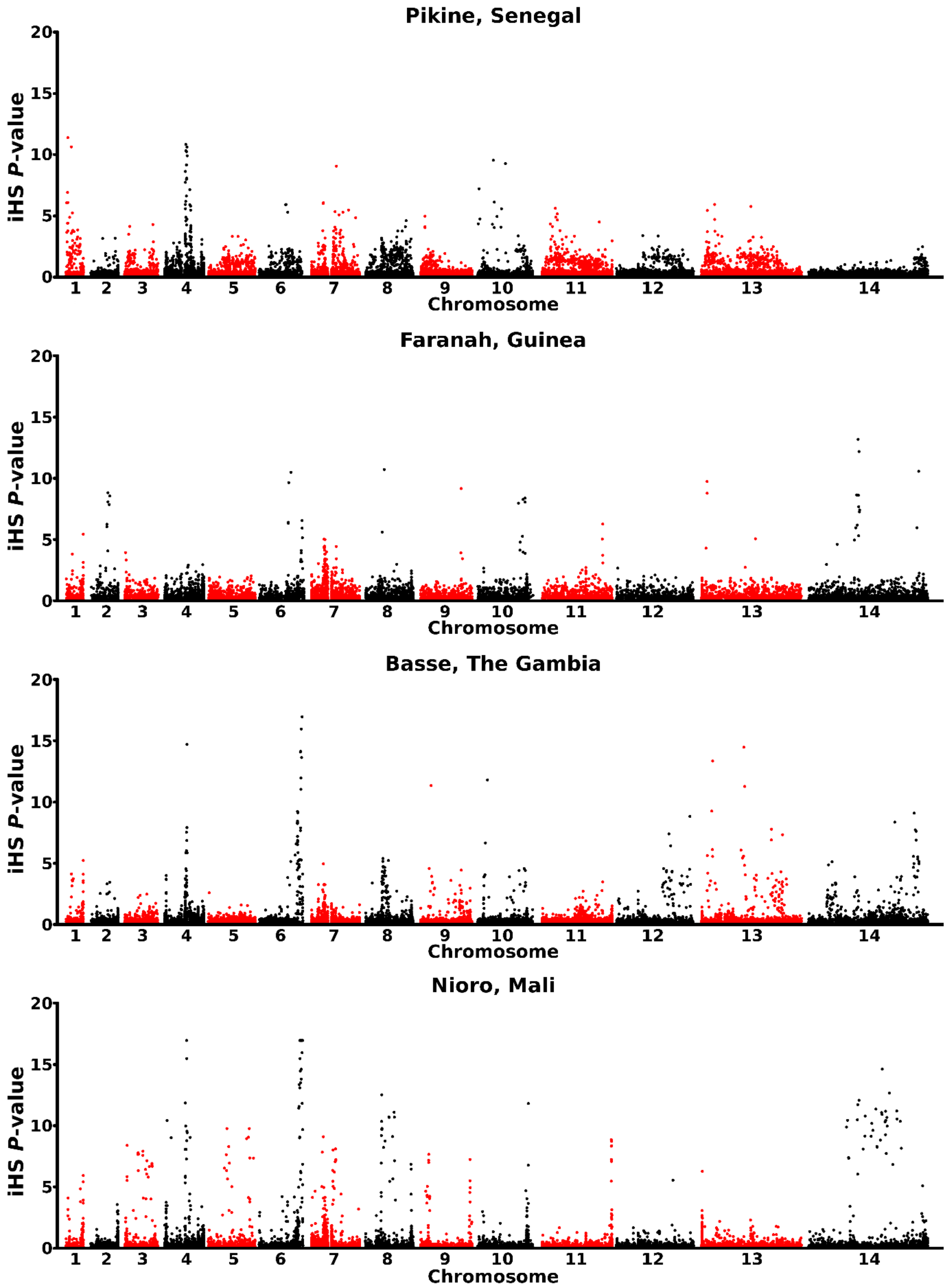

Fig. S1. Genome-wide scan of Integrated Haplotype Scores (iHS) for each of the four newly sampled populations in West Africa (Pikine N = 59 clinical isolates analysed, Faranah N = 24, Basse N = 80, and Nioro = 51). The individual values for all SNPs in each of the populations are given in Datafile S1.

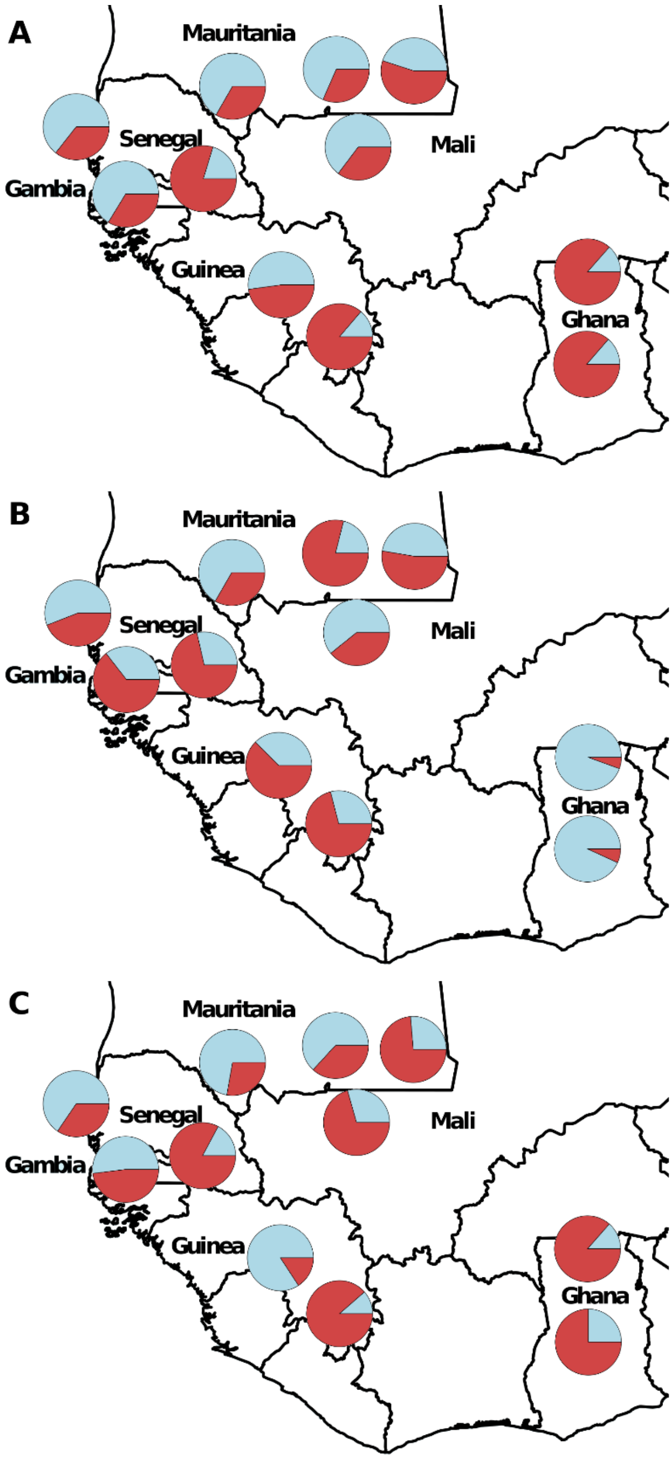

Fig. S2. Allele frequency distributions of the top SNPs in the high F_{ST} windows (apart from the region on chromosome 9 with the genome-wide highest F_{ST} SNP shown in Fig 3). **A.** Chromosome 4 position 1,137,593 (208bp downstream of *hyp15* gene PF3D7_0425200). **B.** Chromosome 7 SNP position 404,407 (within the drug resistance gene *crt*). **C.** Chromosome 8 SNP position 703,102 (400bp upstream of gene PF3D7_0814600).

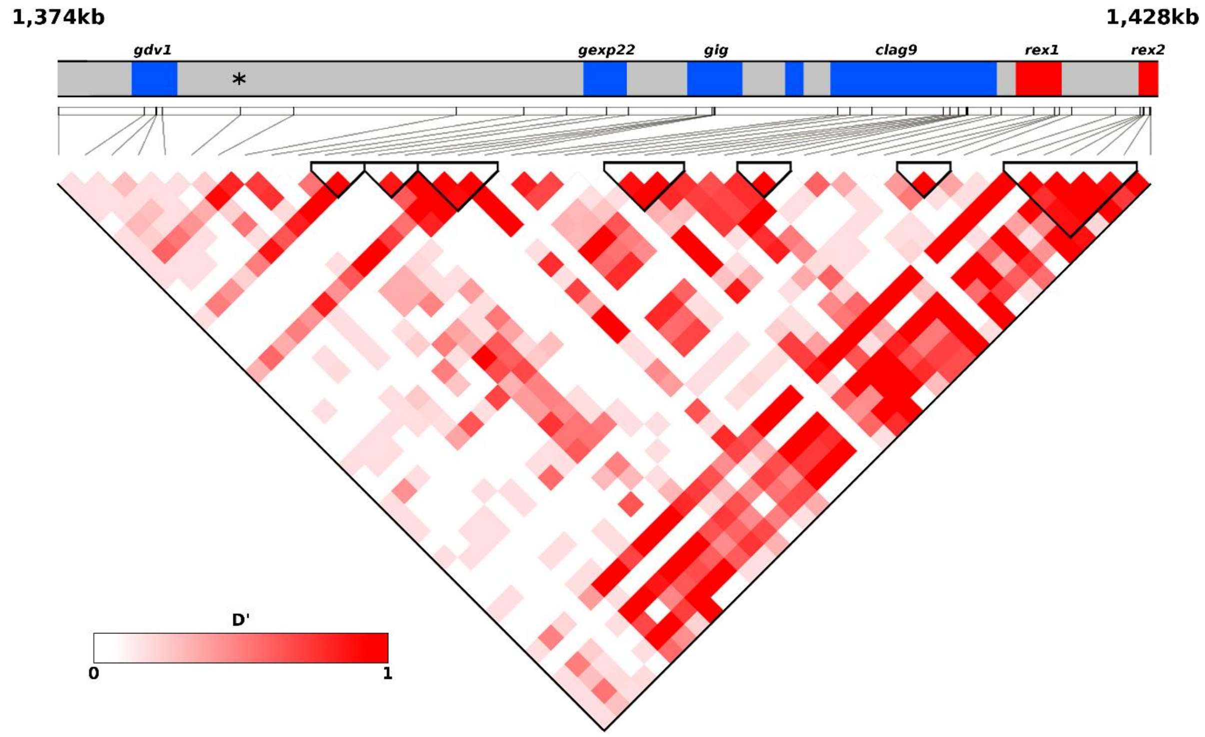

Fig. S3. Extended linkage disequilibrium (LD) across a 54kb region of chromosome 9, for 32 SNPs with a minor allele frequency > 0.1 (data were analysed for predominant single clone infections with F_{WS} > 0.95). Bright red indicates high level of pairwise LD, with D' = 1 and LOD ≥ 2. Shades of pink indicate varying levels of D' along with LOD ≥ 2. Strong LD was observed for many SNPs in the region including the highest F_{ST} SNP at position 138344 (position shown with an asterisk).

Fig. S4.

3D7 sequence:

tattatgcctttaatatacacactaatattttttggcaatatccatatataatattttaattgtgtgcacattaatattataaaatcataatataatttatatattaatatagtatcaaaaggggggaaaaaa
atatatatatatttaataaaataatgtgaaaaaaaagattattcttatatgaacaaataattgttatataaataaaataaaataaaaaaaaaaaacatatatatatatatatatatatatata ttatgat
gttacgtgtttattttaaataagtgctcttttatataaaccgaatggactatgaaaattaattaagttgcgaatattataaataaaaaaatatatatcatatatatatataattacatggaacatttagatata
atttaatacagtgtaataattataaatataattgtataaatattattaatacttaatgaacacatgcacatatacatagtatggaatttgttttaaatcaaatacgttcatccttattttctaaa ttttcaatattt
ttcatattattttttttttattttgtgttgttttattttctggacaactttattgtaatattatttaaaaaaatatataacacgaattaattcttttaaaagaagtgaagcatcgtctatgtatttatattaataaattt
gtttattcttattaaataaaagcataaagtcattgattattatttcaaacatgtaaaatatattattttttattattttatattatgaactacttaataaataatatatatataataaaatatttt catccatatgta
atataacactgtttttgtaccgcattataaagaactatgtaattactccatatatattgtatatattgtatatattatttaagttcatggtattactattatacaagaatataaacaatgttaatgaactatgctg
taaaaaatataaaataatatcaaaaataaataaataaaaaaaaaaaaaaaaaaaaaaaaaaaaaaaggtttatataaattataataaatttaataaagaaacatatatatatttatatcatttcct atat
atacacttatacaatatatgtattattttgaaatagttataaggtatatatcacgtaggttgttataatatatatatatatatatatattattatatatgaataaatatttaatacaatatgataatattaaaaa
atatggatggaaattatcatataatatctgtaattaaaaggatataaatataaacacaaataagtaataataataataaattaaaaaaaatatatataataaataccatattcgattattatttt gcctttttt
ttttttttttctgttgcatgtttttattatggatatttattattttataatataaatatatattagtgtaaaaaatacgcatgtgtacaaatataataatattgtatgtatgtattcatataatatatattttca attt
tttattgaaaataaaatatatatatatatatatatatatattatgatagagtcattcctcacacacacgctatatgaatagtataaatatattatattactttataatataccaccttatgatatgcaagactat
aaataaaataagcttatataataagatatgcatgcacatgtaaaatgaataatatatatatatatatatataaatctaaaagtaaatatatcagtgtattaacacatgtgcaaaatgtattgttaca tatatat
atatatatattatatatatgtttatatgaatttaaaaaattatattaaaatatatgttaccttttacaaccatattaaaatataataattttttttgtatatattgcggatattac tagagataaaataaaaaaa
aggaaatacatgcatctacatatatatatatatatatatatatatatattaatatatgtaaatcaataagtattttttttacaataaaataaaaatataaataacatataatacatatacggttt atgtatgttt
gcttaatattatataaatatatatatatatatatatatatatatatatatataatttatatgtatacatgtgtgtgtattatgtacgtttaatttatgaaat ataaatatggtagcatctcattatataaatttattt
aatatttttcacatactcatatatatattttatatacctttcatgtatatatattataatattatttattaatatatgtttttaaaatgtgcataataaatttgacttatttaaaaaggtaat ataaaaatataaa
taattaattaaataaaaacatataatatatatatatatatatatatatatatatgaataatgtgtgtttctgttttttttta tatgtagtgttattattcaatgaaatatataaaactaacaattataagatttgtt
cttaatatatatatatatatataattagatacatttttattgtttttttatttttattattattgctattttaatttttttttcttttttttttttttttcattaaaaattgtgttatactatagaac aagcatagatattaatta
catgaatatatttattaactatgataaatttatatgtatgtatacatatgtgtatatttcatcgtttgttatattttatgattatatttattatgtcttataagaagtcacagcttcatgtattatttagtgtgatgta
aatataagagattgatataaatatgcttaaatatattatcctaaaaaatgaaaaaaattaaagaaaaattaaaataaaaataaaaaaaaaaaaaaaaaaaaaataaaaacatatctaattagata tat
ttatatatatatatatataataagataaaatcttatagtggttaaccttatttatttcactgaataataacactacatataaaaaaaaaaaaaaaaaaacagaaacacatattattcatatatatatatatatt
tatttatttatttatttatttatttatttatttatgtatatttatttagctttatctttatttttgtataatgtattttttaggtacaacataaaatatatacacatcatataaacgatag tattattattaaaata
ataggacatatatatgtgacattattattatatatatatatatatattatatacacataatgctagacacgtacgtacatttgtgaggttatcattttttttttttttttttttttattatacatttctttacttatata
ttttatatttccatagtatttcaaatgaatacataattatacatatgaaaaaaaaaaaaaaaataataaaataatgagacacatggtttaatatattcctatgaattwatataacatataaatata tataata
tgtatatatatatatatatatatatatatatatatatatatatatatatcatctataatttattaaaacatattttaaaggtcttcaacgtggaaacatataaaggacatataagtatacatttatatgaatata
tttataatatatatatattatatatatagatatatatatatatatatatatatatatatatatatatatagatatatatagatatatatacatatgtggaaactttatttttaaatatgatata cgaaatattaat
atggagaaatctttgtatggtcttttaatatttttatttttaataggatagaatataaacgaataaataaataaatatatataatatatataatgtatatattatgaatacatgaaaaaggttga caattcaaa
taaaatcatactttcacaaatgaagtttgttcttgcctttttttttttaaactttcaatataaggttacatattttattcatcgaaattatttcttatatgttcattgaagaatggat atatatatatatatatattt
acatatatttatatttatatatatatttacatatatttatatttatatatatatttatatatttatgttcatttataatcatttttatgatatatataagggaggaagtgtgaaccaatttttttt ttttaaaagacattt
taaaattatatgcatataatatattattaatagaaattaaatatacataatatagtatatataatatatatataatatatatatatatatatatatatatatataatataaacattgatgtctgaaatttaca
tattaaatatatattatatatatatatatttatagttatggttataattatacattatatatttatatatattttaaaaaattatattatattttacagtttaactttttatttcctaaatta tataaagaatatgat
ttaacataaaaaatgaaattaagtaattatattatatatataataatatacatacatattttgtttttctctttattttttttttcatcatttatttttgtttttcgccatatatatatatatatatatatataatatatat
atattatacttaggaatatcttttaaagtcatatgaatttacatacagcaaaaaaaattatacggaaaaaaaaaaaaaaaaaaaaagaagaatatcttctaatatattttcattctctttttttttt ttcttttttt
tctttcttccttttttccttactttcttacttaccttttttctgggttttttgtttctttattggttttttttttttttttttttttcttcttttcttcttttcttcttttcttcgttttgatttcctttatctggtttccttttttattttcct
caatgtactaaattttttgtatcttttaaaaaataattctgaaaaaaaaaaaaaaatatatatattattacataatatattaacatattcttttattataaaagaattttttttcatgttttttttt ttttttttttttttt
tttgtacattattatatattatttttatgtcctcatattgattatttttatttaacctttaaatatatatatattataaatatataattaatatttttaataatttaaatattatatatatatatatgtattttttttttaat
ttttttaattttttttttttttttttttgtttttttaatttattatatgatataaataaaaatatatgtatataattttatacatatttctattttcatacgatattttatattacatatggataata tatgatatttattttgaa
atattatacatataaaatatatatgtaatttagtatggtattataaagtatctaaaataaaatatgatgaaataatatattattagaaaagttattatatatataaatatatatatatatatata tatgtttatt
aaaagaaaagaatacaaaatttttatataaaccatataaacataaaaaaaatataaaataataataaataaataaataaatatatatatatatatatatttatatatttatat atgtgctataatttgatgaat
atgcacatatataaatatgtgtgttattctatttcataa

(Figure continues on next page)

Dd2 sequence:

tattatgcctttaatatacacactaatattttttggcaatatccatatataatattttaattgtgtgcacattaatattataaaatcataatataatttatatattaatatagtatcaaaaaggggggaaaaaa
aaatatatatatttaataaaataatgtgaaaaaaaagattattcttatatgaacaaataattgttatataaataaaataaaataaaaaaaaaaaacatatatatatatatattatgatgttacgtgtttattt
taaataagtgctcttttatataaaccgaatggactatgaaaattaattaagttgcgaatattataaataaaaaaaatatatatcatatatatatataattacatggaacatttagatataatttaatacagtgt
aataattataaatataattgtataaatattattaatacttaatgaacacatgcacatatacatagtatggaatttgttttaaatcaaatacgttcatccttattttctaaattttcaatatttttcatattattttttt
ttttattttgtgttgttttattttctggacaactttattgtaatattatttaaaaaaatatataacacgaattaattcttttaaaagaagtgaagcatcgtctatgtatttatattaataaatttgtttattcttatta
aataaaagcataaagtcattgattattatttcaaacatgtaaaatatattatttttattattttatattatgaactacttaataaataatatatatataataaaatattttcatccatatgtaatataacactgt
ttttgtaccgcattataaagaactatgtaattactccatatatattatatatattgtatatattatttaagttcatggtattactattatacaagaatataaacaatgttaatgaactatgctgtaaaaaatata
aaataatatcaaaaataaataaataaaaaaaaaaaaaaaaaaaaaaaaaaaaaaaaaaaaggtttatataaattataataaatttaataaagaaacatatatatatttatatcatttcctatatataca
cttatacaatatatgtattattttgaaatagttataaggtatatatcacgtaggttgttataatatatatatatatatatatatatatatatatatatatttatatttatatttatttacttttattttacataatttcatt
tttttatattatatctgaatataggtatgaagtttaaaaatatgttaaaatataagaaaaaaaaaaaaaaatatatatatatatatatataatgataataacggaagaataaattattatattaattataaaa
aatatataatcttgctatattatatatatatatataatacaatacaataccgtttgtgtaatttatttaatattaatccattaagaatatatataaatatataattaatataaatccttatcttacgtaaaataat
aaataaaccccaaattgagtaatacatttattcatcttatattaaaaataaaaaagatcataaacacacatacacacacacacatacacatatatatatatatatatatatatatatatatataatacatat
cctttgtgtacaattttatatattataaagttctttatataaaatttgtatgttttatatgagcatatatatttttttatattaatattttatattaatataagataaactggaatgaacgcaaaatatatttaataa
aaaaatataataaaaatactataaattatttggatatgtatattataatgcacacacatatacacatacatgcatatatacacaaatatatttatatatatattttttatatatttatatatattatcacatgata
cataaatttaatgtcaattcgtttttttcaatatgtatatttttgatttatcgataatctcatataattaatggaatcagaaaatataagatgaatattaaattatgataaaagatatataagtagaaataata
ataaagaagtatatatatatatatatatatatatatatatatatataaagtaaatataaagaataaatctgtcttgcatatataattaaatatatttatatatcatataaaatcattctgttaaattagcgtgct
tgaattttacgcaaccatatctatatatatatatatatatatataataatgataaatagcgatatataatataaaaataataaattcattacatatctaaatattttcatttatacacatacgatacaccttttata
agaaatattaagtataaaatagatattattattatgattacatatatatatatatatatatatatacttacacatttatataaattatatgacataatttatacaatatatataatatataaagtaattgtaaag
atatatatgttaatattttattattataaatcataattatttttatattacacaagaacatgaatccgataaatacaatttatcgtagaaatatatctattattaaaaataaaacactattacataaataaatat
aaatgtatataaagaacaaattatttcttcagactatatatatagatatatatatatatatatatatatatatatatatatatatattattatatatgaataaatatttaatacaatatgataatattaaaaaatatggat
ggaaattatcatataatatctgtaattaaaaggatataaatataaacacaaataagtaataataataataaattaaaaaaaatatatataataaataccatattcgattattattttgcctttttttttttttttt
tttctgttgcatgtttttattatggatatttattattttataatataaatatatattagtgtaaaaaatacgcatgtgtacaaatataataatattgtatgtatgtattcatataatatatattttcaatttttattg
aaaataaaatatatatatatatatatatatatatatatatatatatattatgatagagtcattcctcacacacacgctatatgaatagtataaatatattatattactttataatataccaccttatgatatgca
agactataaataaaataagcttatataataagatatgcatgcacatgtaaaatgaataatatatatatatatatatatatatataaatctaaaagtaaatatatcagtgtattaacacatgtgcaaaatgtattgt
tacatatatatatatatatattatatatatgtttatatgaatttaaaaaattatattaaaatatatgttaccttttacaaccatattaaaatataataattttttttgtatatattgcggatattactagagataaa
ataaaaaaaaggaaatacatgcatctacatatatatatatatatatatatatatatattaatatatgtaaatcaataagtatttttttacaataaaataaaaatataaataacatataatacatatacggttta
tgtatgtttgcttaatattatataaatatatatataatttatatgtatacatgtgtgtgtattatgtacgtttaatttatgaaatataaatatggtagcatctcattatataaatttatttaatatttttcacatactc
atatatatattttatataccttttcatgtatatatattataatattatttattaatatatgtttttttaaaatgtgcataataaatttgacttatttaaaaaggtaatataaaaatataaataattaattaaataaaa
acatataatatatatatatatatatatatatatatatatgaataatgtgtgtttctgtttttttttatatgtagtgttattattcaatgaaatatataaaactaacaattataagatttttcttaatatatatatat
atataattagatacatttttattgtttttttatttttattattattgctattttaatttttttcttttttttttttttttcattaaaaattgtgttatactatagaacaagcatagatattaattacatgaatatatttatt
aactatgataaatttatatgtatgtatacatatgtgtatatttcatcgtttgttatattttatgattatatttattatgtcttataagaagtcacagcttcatgtattatttagtgtgatgtaaatataagagattga
tataaatatgcttaaatatattatcctaaaaaatgaaaaaaattaaagaaaaattaaaataaaaataaaaaaaaaaaaaaaaaaaaaaataaaaacatatctaattagatatatttatatatatatata
tataataagataaaatcttatagtggttaaccttatttatttcactgaataataacactacatataaaaaaaaaaaaaaaaaaaacagaaacacatattattcatatatatatatatatttatttatttatttat
ttatttatgtatatttatttagctttatctttatttttgtataatgtattttttaggtacaacataaaatatatacacatcatataaacgatagtattattattaaaataataggacatatatatgtgacattattat
tatatatatatatatatatataatataaacattgatgtctgaaatttacatattaaatatatattatatatatatatatttatagttatggttataattatacattatatatttatatatatttttaaaaaattatatta
tattttacagtttaactttttttatttcctaaattatataaagaatatgatttaacataaaaaatgaaattaagtaattatattatatatataataatatacatacatattttgtttttctctttattttttttttcatc
atttatttttgtttttcgccatatatatatatatatatatatatatatatatatatatataatatacttaggaatatcttttaaagtcatatgaatttacatacagcaaaaaaaattatacggaaaaaaaaaaaaaaa
aaaaagaagaatatcttctaatatattttcattctctttttttttttcttttttttctttcttcctttttttccttactttcttacttaccttttttctgggttttttgtttctttattggttttttttttttttttttttttttcttcttttct
tcttttcttcgttttgatttcctttatctggtttcctttttttattttcctcaatgtactaaattttttgtatcttttaaaaaataattctgaaaaaaaaaaaaaaaaatatatatattattacataatatattaacatattct
tttattataaaagaattctttttcatgtttttttttttttttttttttttttttttgtacattattatatattattttttatgtcctcatattgattatttttatttaacctttaaatatatatatatattataaatatataattaata
tttttaataatttaaatattatatatatatatatatgtattttttttttaatttttttaatttttttaattttttttttgtttttttaatttattatatgatataaataaaaatatatgtatataattttatacatatttctattttca
tacgatattttatattacatatggataatatatgatatttattttgaaatattatacatataaaatatatatgtaatttagtatggtattataaagtatctaaaataaaatatgatgaaataatatattattaga
aaagttattatatatataaatatatatatatatatatatgtttattaaaagaaaagaatacaaaatttttataaaccatataaacataaaaaaaaatataaaataataataaataaataaataaatatatat
atatatatatatttatatatttatatatgtgctataatttgatgaatatgcacatatataaatatgtgtgttattctatttcataa

Fig. S4. Primary sequence of *gdv1* 3’-intergenic deletion region including primers for PCR genotyping. Sequences of both allelic deletions are shown separately, starting at position 1394810 of the 3D7 reference chromosome 9 sequence and ending at position 1400323. For the Dd2 alternate type the start and end of the sequences align to the respective positions of the 3D7 reference sequence. Yellow highlights the two allele-specific indel segments, and green indicates positions for the four primers used in the PCR typing of the insert sizes. Primer sequences for the first deleted segment were Chr9-block1-F3 (5’-acactgtttttgtaccgcattataaag-3’) and Chr9-block2-R2 (5’-agcgtgtgtgtgaggaatgactc-3’) producing fragments of 739bp in 3D7 and 2245bp in Dd2. Primer sequences for the second deleted segment were Chr9-block2-F1 (5’-gtcttataagaagtcacagcttcatgt-3’) and Chr9-block3-R2 (5’-acccagaaaaaggtaagtaagaaagt-3’) producing a 2001bp fragment in 3D7 and 974bp in Dd2.

Table S1. European Nucleotide Archive accession numbers for paired-end short-read parasite genome sequences of 284 new *P. falciparum* clinical infection samples from four West African countries. SNP genotype calls were performed within the MalariaGEN 6.0 pipeline and have been added to the MalariaGEN public database (https://www.malariagen.net/projects/p-falciparum-community-project). Within-isolate fixation indices (F_{WS} values comparing to local population diversity) are given for the samples that were included in the SNP analysis (having low data missingness as described in Materials and Methods).

Country: Site	Infection Sample ID	ENA Accession Number	MalariaGEN Reference	Sequence ID	F_{WS} value
Guinea: Faranah	GNV057	ERS433599	1026	PA0280-CW	
Guinea: Faranah	GNV075	ERS433600	1026	PA0281-CW	
Guinea: Faranah	GNV039	ERS433601	1026	PA0283-CW	
Guinea: Faranah	GNV112	ERS433602	1026	PA0284-CW	0.84
Guinea: Faranah	GNV123	ERS433603	1026	PA0285-CW	
Guinea: Faranah	GNV087	ERS433605	1026	PA0288-CW	
Guinea: Faranah	GNV141	ERS340316	1026	PA0289-C	
Guinea: Faranah	GNV141	ERS433606	1026	PA0289-CW	
Guinea: Faranah	GNV062	ERS340317	1026	PA0290-C	0.36
Guinea: Faranah	GNV130	ERS433607	1026	PA0291-CW	0.64
Guinea: Faranah	GNV015	ERS433608	1026	PA0292-CW	
Guinea: Faranah	GNV124	ERS433609	1026	PA0293-CW	
Guinea: Faranah	GNV042	ERS433610	1026	PA0294-CW	
Guinea: Faranah	GNV151	ERS433611	1026	PA0295-CW	
Guinea: Faranah	GNV129	ERS433653	1026	PA0297-CW	0.97
Guinea: Faranah	GNV097	ERS340325	1026	PA0298-C	0.68
Guinea: Faranah	GNV115	ERS433654	1026	PA0299-CW	
Guinea: Faranah	GNV094	ERS340327	1026	PA0300-C	0.49
Guinea: Faranah	GNV085	ERS340329	1026	PA0302-C	0.99
Guinea: Faranah	GNV111	ERS433655	1026	PA0303-CW	0.87
Guinea: Faranah	GNV092	ERS433657	1026	PA0305-CW	0.75
Guinea: Faranah	GNV109	ERS433658	1026	PA0306-CW	0.77
Guinea: Faranah	GNV026	ERS433659	1026	PA0307-CW	0.99
Guinea: Faranah	GNV078	ERS433662	1026	PA0312-CW	
Guinea: Faranah	GNV048	ERS433663	1026	PA0313-CW	
Guinea: Faranah	GNV029	ERS433666	1026	PA0318-CW	0.99
Guinea: Faranah	GNV138	ERS340347	1026	PA0320-C	
Guinea: Faranah	GNV096	ERS433669	1026	PA0321-CW	
Guinea: Faranah	GNV157	ERS433673	1026	PA0325-CW	
Guinea: Faranah	GNV043	ERS433675	1026	PA0327-CW	
Guinea: Faranah	GNV148	ERS433676	1026	PA0328-CW	
Guinea: Faranah	GNV128	ERS433677	1026	PA0329-CW	0.97
Guinea: Faranah	GNV090	ERS433678	1026	PA0330-CW	
Guinea: Faranah	GNV137	ERS433679	1026	PA0331-CW	
Guinea: Faranah	GNV103	ERS433680	1026	PA0332-CW	1.00
Guinea: Faranah	GNV031	ERS433682	1026	PA0334-CW	0.74
Guinea: Faranah	GNV070	ERS433683	1026	PA0335-CW	

Guinea: Faranah	GNV069	ERS340364	1026	PA0337-C	0.97
Guinea: Faranah	GNV134	ERS433685	1026	PA0339-CW	0.64
Guinea: Faranah	GNV089	ERS433686	1026	PA0340-CW	
Guinea: Faranah	GNV052	ERS340368	1026	PA0341-C	0.78
Guinea: Faranah	GNV104	ERS433687	1026	PA0342-CW	
Guinea: Faranah	GNV139	ERS433688	1026	PA0343-CW	
Guinea: Faranah	GNV028	ERS433689	1026	PA0344-CW	0.61
Guinea: Faranah	GNV076	ERS433690	1026	PA0345-CW	0.99
Guinea: Faranah	GNV053	ERS433691	1026	PA0346-CW	0.99
Guinea: Faranah	GNV074	ERS433692	1026	PA0347-CW	0.97
Guinea: Faranah	GNV147	ERS340376	1026	PA0349-C	1.00
Guinea: Faranah	GNV116	ERS433693	1026	PA0350-CW	0.84
Guinea: Faranah	GNV063	ERS433694	1026	PA0351-CW	
Guinea: Faranah	GNV132	ERS433696	1026	PA0353-CW	
Guinea: Faranah	GNV158	ERS433697	1026	PA0354-CW	
Guinea: Faranah	GNV080	ERS433699	1026	PA0356-CW	
Guinea: Faranah	GNV143	ERS433700	1026	PA0357-CW	
Guinea: Faranah	GNV150	ERS433701	1026	PA0358-CW	
Guinea: Faranah	GNV203	ERS433705	1026	PA0362-CW	
Guinea: Faranah	GNV202	ERS433706	1026	PA0363-CW	
Guinea: Faranah	GNV160	ERS433707	1026	PA0364-CW	
Guinea: Faranah	GNV086	ERS433711	1026	PA0369-CW	
Mali: Nioro	N011	ERS657636	1134	PM0529-C	1.00
Mali: Nioro	N017	ERS657642	1134	PM0535-C	1.00
Mali: Nioro	N026	ERS657651	1134	PM0544-C	
Mali: Nioro	NO47	ERS739757	1134	PM0565-C	0.95
Mali: Nioro	NO48	ERS739758	1134	PM0566-C	0.99
Mali: Nioro	NO49	ERS739759	1134	PM0567-C	1.00
Mali: Nioro	NO50	ERS739760	1134	PM0568-C	0.83
Mali: Nioro	NO51	ERS739761	1134	PM0569-C	1.00
Mali: Nioro	NO52	ERS739762	1134	PM0570-C	1.00
Mali: Nioro	NO53	ERS739763	1134	PM0571-C	1.00
Mali: Nioro	NO54	ERS739764	1134	PM0572-C	0.83
Mali: Nioro	NO55	ERS739765	1134	PM0573-C	0.99
Mali: Nioro	NO56	ERS739766	1134	PM0574-C	1.00
Mali: Nioro	NO57	ERS739767	1134	PM0575-C	0.68
Mali: Nioro	NO58	ERS739768	1134	PM0576-C	0.83
Mali: Nioro	NO59	ERS739769	1134	PM0577-C	0.55
Mali: Nioro	NO60	ERS739770	1134	PM0578-C	0.89
Mali: Nioro	NO61	ERS739771	1134	PM0579-C	0.95
Mali: Nioro	NO62	ERS739772	1134	PM0580-C	0.98
Mali: Nioro	NO63	ERS739773	1134	PM0581-C	0.79
Mali: Nioro	NO64	ERS739774	1134	PM0582-C	0.99
Mali: Nioro	NO65	ERS739775	1134	PM0583-C	1.00
Mali: Nioro	NO66	ERS739776	1134	PM0584-C	0.72
Mali: Nioro	NO67	ERS739777	1134	PM0585-C	0.99

Mali: Nioro	NO68	ERS739778	1134	PM0586-C	1.00
Mali: Nioro	NO69	ERS739779	1134	PM0587-C	0.70
Mali: Nioro	NO70	ERS739780	1134	PM0588-C	1.00
Mali: Nioro	NO71	ERS739781	1134	PM0589-C	0.85
Mali: Nioro	NO72	ERS739782	1134	PM0590-C	0.99
Mali: Nioro	NO73	ERS739783	1134	PM0591-C	0.85
Mali: Nioro	NO74	ERS739784	1134	PM0592-C	1.00
Mali: Nioro	NO75	ERS739785	1134	PM0593-C	0.99
Mali: Nioro	NO76	ERS739786	1134	PM0594-C	1.00
Mali: Nioro	NO77	ERS739787	1134	PM0595-C	0.99
Mali: Nioro	NO78	ERS739788	1134	PM0596-C	0.59
Mali: Nioro	NO79	ERS739789	1134	PM0597-C	0.83
Mali: Nioro	NO80	ERS739790	1134	PM0598-C	0.89
Mali: Nioro	NO81	ERS739791	1134	PM0599-C	0.55
Mali: Nioro	NO82	ERS739792	1134	PM0600-C	1.00
Mali: Nioro	NO83	ERS739793	1134	PM0601-C	0.99
Mali: Nioro	NO84	ERS739794	1134	PM0602-C	0.99
Mali: Nioro	NO85	ERS739795	1134	PM0603-C	0.99
Mali: Nioro	NO86	ERS739796	1134	PM0604-C	0.99
Mali: Nioro	NO87	ERS739797	1134	PM0605-C	0.99
Mali: Nioro	NO88	ERS739798	1134	PM0606-C	0.99
Mali: Nioro	NO89	ERS739799	1134	PM0607-C	0.94
Mali: Nioro	NO90	ERS739800	1134	PM0608-C	0.67
Mali: Nioro	NO91	ERS739801	1134	PM0609-C	1.00
Mali: Nioro	NO92	ERS739802	1134	PM0610-C	0.99
Mali: Nioro	NO93	ERS739803	1134	PM0611-C	0.98
Mali: Nioro	NO94	ERS739804	1134	PM0612-C	0.98
Mali: Nioro	NO95	ERS739805	1134	PM0613-C	1.00
Senegal: Pikine	PK05/13	ERS657673	1135	QV0002-C	0.78
Senegal: Pikine	PK08/13	ERS657675	1135	QV0004-C	0.99
Senegal: Pikine	PK09/13	ERS657676	1135	QV0005-C	0.99
Senegal: Pikine	PK12/13	ERS657679	1135	QV0008-C	0.99
Senegal: Pikine	PK13/13	ERS657680	1135	QV0009-C	0.84
Senegal: Pikine	PK14/13	ERS657681	1135	QV0010-C	0.78
Senegal: Pikine	PK17/13	ERS657684	1135	QV0013-C	1.00
Senegal: Pikine	PK18/13	ERS657685	1135	QV0014-C	0.99
Senegal: Pikine	PK19/13	ERS657686	1135	QV0015-C	1.00
Senegal: Pikine	PK20/13	ERS657687	1135	QV0016-C	0.99
Senegal: Pikine	PK22/13	ERS657689	1135	QV0018-C	1.00
Senegal: Pikine	PK23/13	ERS657690	1135	QV0019-C	0.96
Senegal: Pikine	PK24/13	ERS657691	1135	QV0020-C	0.99
Senegal: Pikine	PK25/13	ERS657692	1135	QV0021-C	0.99
Senegal: Pikine	PK30/13	ERS657697	1135	QV0026-C	0.99
Senegal: Pikine	PK31/13	ERS657698	1135	QV0027-C	
Senegal: Pikine	PK32/13	ERS657699	1135	QV0028-C	0.80
Senegal: Pikine	PK33/13	ERS657700	1135	QV0029-C	0.90

Senegal: Pikine	PK34/13	ERS657701	1135	QV0030-C	1.00
Senegal: Pikine	PK36/13	ERS657702	1135	QV0031-C	1.00
Senegal: Pikine	PK37/13	ERS657703	1135	QV0032-C	1.00
Senegal: Pikine	PK38/13	ERS657704	1135	QV0033-C	0.99
Senegal: Pikine	PK40/13	ERS657706	1135	QV0035-C	0.99
Senegal: Pikine	PK42/13	ERS657708	1135	QV0037-C	0.99
Senegal: Pikine	PK43/13	ERS657709	1135	QV0038-C	0.99
Senegal: Pikine	PK44/13	ERS657710	1135	QV0039-C	0.99
Senegal: Pikine	PK45/13	ERS657711	1135	QV0040-C	0.99
Senegal: Pikine	PK46/13	ERS657712	1135	QV0041-C	0.61
Senegal: Pikine	PK47/13	ERS657713	1135	QV0042-C	0.98
Senegal: Pikine	PK49/13	ERS657715	1135	QV0044-C	1.00
Senegal: Pikine	PK50/13	ERS657716	1135	QV0045-C	1.00
Senegal: Pikine	PK51/13	ERS657717	1135	QV0046-C	0.99
Senegal: Pikine	PK52/13	ERS657718	1135	QV0047-C	1.00
Senegal: Pikine	PK54/13	ERS657720	1135	QV0049-C	0.99
Senegal: Pikine	PK56/13	ERS657722	1135	QV0051-C	0.99
Senegal: Pikine	PK58/13	ERS657724	1135	QV0053-C	0.99
Senegal: Pikine	PK61/13	ERS657727	1135	QV0056-C	0.99
Senegal: Pikine	PK63/13	ERS657729	1135	QV0058-C	0.99
Senegal: Pikine	PK64/13	ERS657730	1135	QV0059-C	0.91
Senegal: Pikine	PK65/13	ERS657731	1135	QV0060-C	0.99
Senegal: Pikine	PK66/13	ERS657732	1135	QV0061-C	0.99
Senegal: Pikine	PK67/13	ERS657733	1135	QV0062-C	1.00
Senegal: Pikine	PK69/13	ERS657735	1135	QV0064-C	0.99
Senegal: Pikine	PK70/13	ERS657736	1135	QV0065-C	1.00
Senegal: Pikine	PK72/13	ERS657738	1135	QV0067-C	1.00
Senegal: Pikine	PK74/13	ERS657740	1135	QV0069-C	0.99
Senegal: Pikine	PK75/13	ERS657741	1135	QV0070-C	0.99
Senegal: Pikine	PK76/13	ERS657742	1135	QV0071-C	0.99
Senegal: Pikine	PK77/13	ERS657743	1135	QV0072-C	0.99
Senegal: Pikine	PK78/13	ERS657744	1135	QV0073-C	0.48
Senegal: Pikine	PK79/13	ERS657745	1135	QV0074-C	0.49
Senegal: Pikine	PK80/13	ERS657746	1135	QV0075-C	0.91
Senegal: Pikine	PK81/13	ERS657747	1135	QV0076-C	0.99
Senegal: Pikine	PK82/13	ERS657748	1135	QV0077-C	1.00
Senegal: Pikine	PK83/13	ERS657749	1135	QV0078-C	1.00
Senegal: Pikine	PK85/13	ERS657751	1135	QV0080-C	0.99
Senegal: Pikine	PK86/13	ERS657752	1135	QV0081-C	0.99
Senegal: Pikine	PK87/13	ERS657753	1135	QV0082-C	0.90
Senegal: Pikine	PK88/13	ERS657754	1135	QV0083-C	0.81
Senegal: Pikine	PK89/13	ERS657755	1135	QV0084-C	1.00
The Gambia: Basse	GNV_002	ERS728719	1136	PA0370-C	0.83
The Gambia: Basse	GNV_003	ERS728720	1136	PA0371-C	1.00
The Gambia: Basse	GNV_004	ERS728721	1136	PA0372-C	0.78
The Gambia: Basse	GNV_005	ERS728722	1136	PA0373-C	

The Gambia: Basse	GNV_006	ERS728723	1136	PA0374-C	1.00
The Gambia: Basse	GNV_007	ERS728724	1136	PA0375-C	0.53
The Gambia: Basse	GNV_008	ERS728725	1136	PA0376-C	0.99
The Gambia: Basse	GNV_009	ERS728726	1136	PA0377-C	1.00
The Gambia: Basse	GNV_012	ERS728727	1136	PA0378-C	0.68
The Gambia: Basse	GNV_013	ERS728728	1136	PA0379-C	1.00
The Gambia: Basse	GNV_016	ERS728731	1136	PA0382-C	0.78
The Gambia: Basse	GNV_017	ERS728732	1136	PA0383-C	
The Gambia: Basse	GNV_019	ERS728733	1136	PA0384-C	1.00
The Gambia: Basse	GNV_021	ERS728735	1136	PA0386-C	
The Gambia: Basse	GNV_022	ERS728736	1136	PA0387-C	1.00
The Gambia: Basse	GNV_023	ERS728737	1136	PA0388-C	0.99
The Gambia: Basse	GNV_024	ERS728738	1136	PA0389-C	0.89
The Gambia: Basse	GNV_026	ERS728740	1136	PA0391-C	1.00
The Gambia: Basse	GNV_027	ERS728741	1136	PA0392-C	0.99
The Gambia: Basse	GNV_028	ERS728742	1136	PA0393-C	0.99
The Gambia: Basse	GNV_029	ERS728743	1136	PA0394-C	0.93
The Gambia: Basse	GNV_030	ERS728744	1136	PA0395-C	0.99
The Gambia: Basse	GNV_031	ERS728745	1136	PA0396-C	1.00
The Gambia: Basse	GNV_032	ERS728746	1136	PA0397-C	0.69
The Gambia: Basse	GNV_033	ERS728747	1136	PA0398-C	1.00
The Gambia: Basse	GNV_034	ERS728748	1136	PA0399-C	0.53
The Gambia: Basse	GNV_035	ERS728749	1136	PA0400-C	1.00
The Gambia: Basse	GNV_036	ERS728750	1136	PA0401-C	1.00
The Gambia: Basse	GNV_037	ERS728751	1136	PA0402-C	0.99
The Gambia: Basse	GNV_038	ERS728752	1136	PA0403-C	
The Gambia: Basse	GNV_039	ERS728753	1136	PA0404-C	0.56
The Gambia: Basse	GNV_040	ERS728754	1136	PA0405-C	0.96
The Gambia: Basse	GNV_041	ERS728755	1136	PA0406-C	0.98
The Gambia: Basse	GNV_043	ERS728756	1136	PA0407-C	0.99
The Gambia: Basse	GNV_045	ERS728758	1136	PA0409-C	0.99
The Gambia: Basse	GNV_046	ERS728759	1136	PA0410-C	
The Gambia: Basse	GNV_047	ERS728760	1136	PA0411-C	0.99
The Gambia: Basse	GNV_049	ERS728762	1136	PA0413-C	0.99
The Gambia: Basse	GNV_050	ERS728763	1136	PA0414-C	0.97
The Gambia: Basse	GNV_054	ERS728766	1136	PA0417-C	1.00
The Gambia: Basse	GNV_056	ERS728768	1136	PA0419-C	0.99
The Gambia: Basse	MVS_004	ERS728772	1136	PA0423-C	0.99
The Gambia: Basse	MVS_005	ERS728773	1136	PA0424-C	0.99
The Gambia: Basse	MVS_006	ERS728774	1136	PA0425-C	
The Gambia: Basse	MVS_012	ERS728780	1136	PA0431-C	
The Gambia: Basse	K0070707	ERS788227	1137	PA0534-C	0.50
The Gambia: Basse	K0344029	ERS788234	1137	PA0537-C	0.99
The Gambia: Basse	K0333902	ERS788243	1137	PA0540-C	0.99
The Gambia: Basse	K0101006a	ERS788246	1137	PA0541-C	0.66
The Gambia: Basse	K0364305b	ERS788250	1137	PA0542-C	0.83

The Gambia: Basse	K0252704	ERS788254	1137	PA0544-C	0.87
The Gambia: Basse	J0242506a	ERS788267	1137	PA0550-C	1.00
The Gambia: Basse	J0202102	ERS788269	1137	PA0551-C	0.99
The Gambia: Basse	J0232410	ERS788276	1137	PA0554-C	0.99
The Gambia: Basse	K0405011	ERS788280	1137	PA0558-C	0.99
The Gambia: Basse	K0050517	ERS788283	1137	PA0561-C	
The Gambia: Basse	K006017	ERS788284	1137	PA0562-C	0.99
The Gambia: Basse	K0344027	ERS788285	1137	PA0563-C	0.99
The Gambia: Basse	K0374411	ERS788286	1137	PA0564-C	0.75
The Gambia: Basse	K0222243	ERS788294	1137	PA0572-C	1.00
The Gambia: Basse	J0242515	ERS788297	1137	PA0575-C	0.99
The Gambia: Basse	J0131406	ERS788298	1137	PA0576-C	0.90
The Gambia: Basse	K0263104B	ERS788299	1137	PA0577-C	1.00
The Gambia: Basse	K0202010	ERS788304	1137	PA0582-C	
The Gambia: Basse	K0252717	ERS788307	1137	PA0585-C	
The Gambia: Basse	K0171704	ERS788308	1137	PA0586-C	0.99
The Gambia: Basse	K0405012	ERS788315	1137	PA0593-C	
The Gambia: Basse	K0161619	ERS788316	1137	PA0594-C	
The Gambia: Basse	K0252817	ERS788319	1137	PA0597-C	0.81
The Gambia: Basse	K0263104	ERS788321	1137	PA0599-C	1.00
The Gambia: Basse	F0627701	ERS788322	1137	PA0600-C	
The Gambia: Basse	F0445603	ERS788325	1137	PA0603-C	
The Gambia: Basse	F0455808	ERS788326	1137	PA0604-C	0.87
The Gambia: Basse	F0708703	ERS788327	1137	PA0605-C	
The Gambia: Basse	F0060808	ERS788328	1137	PA0606-C	
The Gambia: Basse	F0152011	ERS788332	1137	PA0610-C	
The Gambia: Basse	E0060808	ERS788333	1137	PA0611-C	
The Gambia: Basse	E0081011	ERS788334	1137	PA0612-C	
The Gambia: Basse	F0314202	ERS788335	1137	PA0613-C	
The Gambia: Basse	E0081103	ERS788337	1137	PA0615-C	
The Gambia: Basse	PG001	ERS788339	1137	PA0617-C	
The Gambia: Basse	PG002	ERS788340	1137	PA0618-C	1.00
The Gambia: Basse	PG003	ERS788341	1137	PA0619-C	0.48
The Gambia: Basse	PG004	ERS788342	1137	PA0620-C	0.52
The Gambia: Basse	PG005	ERS788343	1137	PA0621-C	
The Gambia: Basse	PG006	ERS788344	1137	PA0622-C	1.00
The Gambia: Basse	PG007	ERS788345	1137	PA0623-C	
The Gambia: Basse	PG009	ERS788347	1137	PA0625-C	0.74
The Gambia: Basse	PG010	ERS788348	1137	PA0626-C	0.99
The Gambia: Basse	PG011	ERS788228	1137	PA0627-C	0.84
The Gambia: Basse	PG012	ERS788230	1137	PA0628-C	0.70
The Gambia: Basse	PG013	ERS788231	1137	PA0629-C	0.67
The Gambia: Basse	PG014	ERS788233	1137	PA0630-C	1.00
The Gambia: Basse	PG015	ERS788235	1137	PA0631-C	0.92
The Gambia: Basse	PG017	ERS788238	1137	PA0633-C	1.00
The Gambia: Basse	PG018	ERS788239	1137	PA0634-C	

The Gambia: Basse	PG019	ERS788241	1137	PA0635-C	
The Gambia: Basse	PG020	ERS788242	1137	PA0636-C	0.61
The Gambia: Basse	PG021	ERS788244	1137	PA0637-C	0.75
The Gambia: Basse	PG022	ERS788245	1137	PA0638-C	0.70
The Gambia: Basse	PG023	ERS788247	1137	PA0639-C	1.00
The Gambia: Basse	PG025	ERS788248	1137	PA0640-C	
The Gambia: Basse	PG029	ERS788249	1137	PA0641-C	0.65
The Gambia: Basse	PG031	ERS788251	1137	PA0642-C	1.00
The Gambia: Basse	PG032	ERS788253	1137	PA0643-C	1.00
The Gambia: Basse	PG035	ERS788255	1137	PA0644-C	
The Gambia: Basse	PG036	ERS788256	1137	PA0645-C	
The Gambia: Basse	PG040	ERS788262	1137	PA0648-C	
The Gambia: Basse	PG042	ERS788264	1137	PA0649-C	0.98
The Gambia: Basse	PG056	ERS788268	1137	PA0651-C	
The Gambia: Basse	K0030302	ERS788272	1137	PA0653-C	
The Gambia: Basse	K0242513	ERS788274	1137	PA0654-C	
The Gambia: Basse	K0333914	ERS788275	1137	PA0655-C	1.00

Table S2. Genotypes of the *gdv1* 3'-intergenic insertion-deletion polymorphism in 13 culture adapted laboratory lines of *P. falciparum* using a PCR assay.

Parasite Line	Putative geographical origin	Chromosome 9 dimorphic indel type
NF54	Africa (imported to Europe)	3D7-type
3D7	Africa (clone of NF54)	3D7-type
Dd2	Southeast Asia	Dd2-type
HB3	Central America	Dd2-type
RO33	Africa	Dd2-type
Wellcome	Origin unclear*	Dd2-type
FCC2	Southeast Asia	Dd2-type
T9/94	Southeast Asia	Dd2-type
FCR3	Origin unclear *	Dd2-type
W2	Southeast Asia	Dd2-type
Palo Alto	Africa	3D7-type
7G8	South America	Dd2-type
D6	Africa	3D7-type

* The Wellcome and FCR3 isolates are nominally supposed to have originated from West Africa over 40 years ago but genomic analyses indicate non-African genotypes typical of Southeast Asian parasites, likely due to historic culture contamination.

Positions and sequences of the alternative allelic segments are shown in Fig S4.
